# Photothermal effective CeO_2_NPs combined in thermosensitive hydrogels with enhanced antibacterial, antioxidant and vascularization performance to accelerate infected diabetic wound healing

**DOI:** 10.1093/rb/rbad072

**Published:** 2023-09-01

**Authors:** Zexiang Zheng, Xing Yang, Min Fang, Jinhuan Tian, Shuyun Zhang, Lu Lu, Changren Zhou, Changpeng Xu, Yong Qi, Lihua Li

**Affiliations:** College of Chemistry and Materials Science, Engineering Research Center of Artificial Organs and Materials, Jinan University, Guangzhou 511486, China; College of Chemistry and Materials Science, Engineering Research Center of Artificial Organs and Materials, Jinan University, Guangzhou 511486, China; College of Chemistry and Materials Science, Engineering Research Center of Artificial Organs and Materials, Jinan University, Guangzhou 511486, China; College of Chemistry and Materials Science, Engineering Research Center of Artificial Organs and Materials, Jinan University, Guangzhou 511486, China; Guangdong Second Provincial General Hospital, Postdoctoral Research Station of Basic Medicine, School of Medicine, Jinan University, Guangdong 510632, PR China; College of Chemistry and Materials Science, Engineering Research Center of Artificial Organs and Materials, Jinan University, Guangzhou 511486, China; College of Chemistry and Materials Science, Engineering Research Center of Artificial Organs and Materials, Jinan University, Guangzhou 511486, China; Department of Orthopaedics, Guangdong Second Provincial General Hospital, Faculty of Medical Science, Jinan University, Guangzhou 510317, China; Department of Orthopaedics, Guangdong Second Provincial General Hospital, Faculty of Medical Science, Jinan University, Guangzhou 510317, China; College of Chemistry and Materials Science, Engineering Research Center of Artificial Organs and Materials, Jinan University, Guangzhou 511486, China; Guangdong Second Provincial General Hospital, Postdoctoral Research Station of Basic Medicine, School of Medicine, Jinan University, Guangdong 510632, PR China; Department of Orthopaedics, Guangdong Second Provincial General Hospital, Faculty of Medical Science, Jinan University, Guangzhou 510317, China

**Keywords:** photothermal hydrogel, sodium alginate, cerium dioxide nanoparticle, gelatin, infected diabetic wound healing

## Abstract

Chronic diabetic wound healing remains a formidable challenge due to susceptibility to bacterial infection, excessive oxidative stress, and poor angiogenesis. To address these issues, a sodium alginate (SA) based photothermal hydrogel dressing with multifunction was fabricated to facilitate wound treatment. Ceria nanoparticles (CeO_2_NPs) was synthesized, and their antibacterial performance by near-infrared light triggered photothermal effects was first studied and verified in this work. In addition, to release CeO_2_NPs to achieve antioxidation and pro-vascularization, thermosensitive gelatin (Gel) was utilized to embed the nanoparticles in advance and then composited in SA hydrogel networks. SA network was finally strengthened by acid soaking to form partially crystalline regions to act as natural crosslinkers. Results showed that the Gel/SA/CeO_2_ hydrogel displayed temperature-responsive release of CeO_2_NPs, significant antibacterial and antioxidative activity, as well as the ability to remove without injury and promote infected diabetic wound healing with low cytotoxicity, according to antibacterial investigations, cell studies, and *in vivo* animal studies. This research offers not only a successful method for quickening the healing of diabetic wounds but also a fresh approach to the general use of CeO_2_NPs.

## Graphical Abstract

**Figure:**
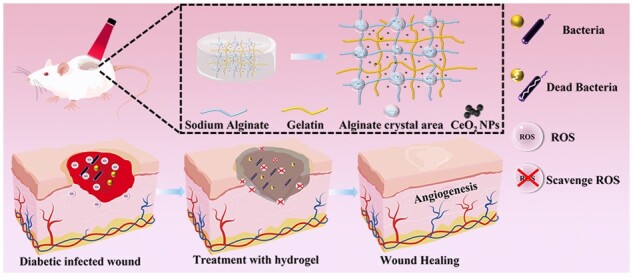

## Introduction

Diabetes mellitus, characterized by hyperglycemia [1], is an endocrine system and metabolic disease and one of the top ten chronic diseases in the world [2]. It is well known that chronic diabetic wounds have become a major challenge to healthcare systems worldwide because of the high rates of morbidity, death, and recurrence [3–5]. Different from other common wounds, chronic diabetic wounds are more susceptible to bacterial infection [6], blocked angiogenesis [7], and excessive accumulation of ROS [8], etc., due to the complex wound microenvironment, which makes the wound difficult to heal [9]. Consequently, the development of a multifunctional dressing with efficient sterilization, induction of angiogenesis, and removal of excess ROS to reduce oxidative stress is of great significance for the treatment of chronic diabetic wounds.

Hydrogels have been widely regarded as ideal dressing candidates for their 3D structure [10], good permeability [11], excellent biocompatibility [12], and ability to provide a moist environment for wound repair [13], overcoming the limitations of conventional dressings [14]. Gelatin (Gel) is a natural polymer that can be extracted from insoluble collagen by hydrolysis[15]. In the biomedical field, Gel is frequently employed because it has many of the same features as collagen, including high biocompatibility, biodegradability, non-immunogenicity, and the capacity to stimulate cell adhesion and proliferation [16–18]. However, because Gel is composed of random macromolecules and heterogeneous structure, its poor melting point makes it rapidly dissolve at 37°C, which limits its further application [19]. Many researchers use chemical cross-linking agents to increase the Gel’s mechanical strength, but they also have certain negative side effects [20]. Sodium alginate (SA), a natural polymer material extracted from seafood, has excellent properties such as good biocompatibility, bio-adhesion, degradability, hydrophilicity [21], low immunogenicity and price [22], has been widely used in the food industry and biomedical field [23]. In this study, SA was used instead of chemical cross-linking agent to construct a physically cross-linked Gel/SA hydrogel [24], which exhibits good biocompatibility while enhancing thermal stability and mechanical properties.

Photothermal therapy (PTT) has become an effective method to treat drug-resistant bacterial infections and promote tissue regeneration due to its advantages of fewer side effects, minimal systemic toxicity, and less intrusiveness [25]. Previous studies have shown that high temperature can effectively inhibit bacterial growth, and mild high temperature can promote cell proliferation, accelerate wound healing and promote bone regeneration [26]. Because PTT requires relatively high temperatures to kill bacteria [27], it may harm the healthy skin tissue surrounding the wound when was used alone. Therefore, loading photosensitizers into hydrogels can reduce the negative effects on normal tissues while exerting photothermal effects. In addition, temperature changes can control the gel-sol transition of thermosensitive hydrogels to release active factors for intensive therapy [28]. Currently, the majority of PTT-based thermosensitive hydrogels is rather complicated and comprises numerous components that serve various purposes [29]. The difficulty of preparation and the risk of clinical application both rise due to the complicated makeup. Therefore, ‘all-in-one’ nanomaterials with a straightforward manufacturing procedure, inherent antibacterial capabilities, and outstanding photothermal conversion efficiency should be the basis of an optimal hydrogel system combining photothermal antibacterial ability and temperature sensitivity [30].

Many nanomaterials with photothermal conversion properties have been developed, such as Ag [31], MoS_2_ [32], WO_3_ [33], MoO_2_ [34], etc. Unfortunately, despite their good photothermal conversion capabilities, many nanoparticles such as silver are highly toxic to normal cells even at low doses [35]. Besides, as the metal ions are released, their inherent antibacterial activity is greatly reduced, resulting in single use [36]. This limits its further application in human healthcare. Cerium dioxide nanoparticles (CeO_2_NPs), as an important member of the rare earth family, are widely used in industry as polishing agents [37], catalysts [38], corrosion inhibitors [39], and sensors [40]. CeO_2_NPs are also increasingly used in biomedicine for their low toxicity to mammalian cells and unique valence switching mechanism. Studies have shown that, as a good free radical scavenger [6], CeO_2_NPs can effectively regulate ROS [41, 42] and have great potential in the treatment of atherosclerosis [43], arthritis [44] and neurodegenerative diseases [45]. Moreover, some studies have shown that CeO_2_NPs can promote cell proliferation and migration around chronic ulcer wounds [46, 47], promote angiogenesis [48], and have a durable bactericidal effect [49], thereby accelerating wound healing. However, to the best of our knowledge, there is no report on CeO_2_NPs for PTT applications so far.

**Figure 1.**
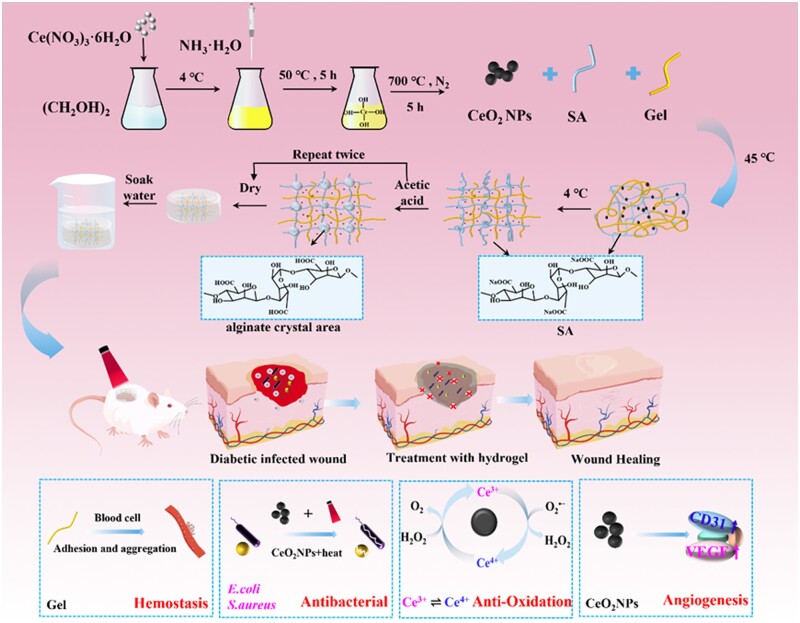
Schematic illustration the preparation and application of gel/SA/CeO_2_ hydrogel.

In this study, CeO_2_NPs with photothermal conversion ability were prepared by a modified precipitation method [50], which not only could maintain ROS balance by utilizing its unique valence state conversion mechanism, but also could be used as photothermal agent to impart photothermal conversion capabilities to hydrogels with the assistance of near-infrared lasers. The schematic illustration of Gel/SA/CeO_2_ hydrogel for diabetic wound healing was shown in Figure 1. First, a physically cross-linked Gel/SA hydrogel was constructed using crystalline domains of alginic acid instead of harmful chemical cross-linking agents. Then, CeO_2_NPs were embedded in the network structure of Gel/SA hydrogel to prepare Gel/SA/CeO_2_ hydrogel dressing. The addition of CeO_2_NPs further enhanced the thermal stability and mechanical properties of Gel/SA, so that the Gel/SA/CeO_2_ hydrogel still maintained a soft gel state under NIR irradiation to accommodate irregular wounds instead of turning into a solution and shedding. Painless peeling can be easily achieved by placing an ice pack on the surface of the hydrogel, thus avoiding secondary damage. This provided a good foundation for the practical application of Gel/SA/CeO_2_ hydrogel dressings. The hydrogel dressing can scavenge ROS, promote the expression of angiogenesis-related factors, effectively sterilization, and remove without injury, thereby accelerating the healing of chronic diabetic wounds.

## Materials and methods

### Materials

Cerium (III) nitrate hexahydrate (99.95%) and Gel (pharmaceutical grade, glue strength ∼240 g Bloom) were purchased from Shanghai Aladdin Bio-Chem Technology Co., Ltd. Ethylene glycol (AR, 98%), Ammonium hydroxide solution (≥28% NH_3_ in H_2_O, electronic grade) and SA (AR, 90%, Mw = 400 kDa, *M*:*G* = 1:1) were purchased from Shanghai Macklin Biochemical Technology Co., Ltd. Phalloidin (iFluor™555) was purchased from Molecular Probes. *Escherichia coli* (ATCC25922) and *Staphylococcus aureus* (ATCC43300) were purchased from Wuhan Warner Biotechnology Co., Ltd, China. AO/EB Live-Dead staining Kit was acquired from Beijing Solarbio Science & Technology Co., Ltd. The Cell Counting Kit-8 Kit (CCK-8) was ordered from Dojindo Molecular Technologies Inc. Human umbilical vein endothelial cells (HUVECs) were purchased from the Cell Bank of Type Culture Collection of the Chinese Academy of Sciences Shanghai, China. All cell culture reagents were purchased from Gibco BRL and other reagents were ordered from SIJIA Biotechnology Co., Ltd.

### Preparation of CeO_2_NPs and characterization of CeO_2_NPs

The method to synthesize CeO_2_NPs was modified according to an earlier publication [50]. Briefly, 2 g of cerium (III) nitrate hexahydrate (Ce (NO_3_)_3_·6H_2_O) was dispersed in 40 ml of ethylene glycol ((CH_2_OH)_2_), and dissolved by ultrasonic for 30 min. Then 15 ml of ammonia water was slowly added, and the reaction was stirred at 50°C for 5 h. At the beginning of the reaction, a pale-yellow precipitate appeared in the solution immediately, then the color of precipitates turned into dark purple, and finally gradually became light yellow. After the reaction was completed, the precipitate was centrifuged (10 000 × rpm, 15 min), washed three times with deionized water and ethanol, freeze-dried, and calcined at a heating rate of 5°C/min at 700°C for 5 h in a nitrogen atmosphere to obtain black CeO_2_NPs.

The crystal structure of CeO_2_NPs was measured by X-ray diffraction (XRD, Miniflex600, Rigaku, Japan). The morphology of CeO_2_NPs was observed by scanning electron microscopy (SEM, Ser.nv.4418, Carl Zeiss, USA). The microstructure of CeO_2_NPs was analyzed by transmission electron microscopy (TEM, PHILIPS TECAI-10, PHILIPS, Netherlands) at an accelerating voltage of 120 kV. The average particle size distribution of the samples was obtained from Image J software. The surface valence components of CeO_2_NPs were determined by X-ray photoelectron spectroscopy (XPS, ESCALAB-250, Thermo, USA). The surface charge of CeO_2_NPs at different pH was tested by Zetasizer (Nano-ZS apparatus, Malvern, UK). The ultraviolet-visible absorption spectrum was measured by UV–vis spectra spectrophotometer (UV-2600, Shimadzu Corporation, Japan).

### Preparation of gel/SA/CeO_2_ hydrogels

To prepare Gel/SA/CeO_2_ hydrogels, 1 g of Gel was first added to 10 ml of deionized water at 45°C for 1 h under continuous stirring. About 0.2 g of SA was then added with stirring to achieve a homogenous solution. Next, a certain amount of CeO_2_NPs powder (0, 20, 40 or 80 mg) was dispersed in the aforementioned solution. Gel/SA, Gel/SA/CeO_2_-0.2%, Gel/SA/CeO_2_-0.4%, and Gel/SA/CeO_2_-0.8% hydrogel precursors were each produced after uniform stirring. Pour each precursor solution into the mold and let it stand at 4°C for 30 min to form initial Gel/SA, Gel/SA/CeO_2_-0.2%, Gel/SA/CeO_2_-0.4%, Gel/SA/CeO_2_-0.8% hydrogel. Subsequently, each group of hydrogels was immersed in 2% acetic acid solution for 10 min, taken out and dried at room temperature for 24 h. Then, the dried hydrogels were immersed in 2% acetic acid solution for 30 min again, taken out and dried at room temperature for 24 h. Finally, the four groups of hydrogels after two acid treatments and drying treatments were immersed in deionized water to obtain Gel/SA, Gel/SA/CeO_2_-0.2%, Gel/SA/CeO_2_-0.4% and Gel/SA/CeO_2_-0.8% hydrogel respectively.

### Characterization of gel/SA/CeO_2_ hydrogels

The 3D porous structures of different hydrogels were observed by SEM, and the elemental composition of the hydrogel samples was verified by EDS mapping. Compression testing of Gel/SA/CeO_2_ was carried out using a universal testing machine (Shimadzuag-1, Shimadzu, Japan) under 25°C. The compression tests of cylindrical hydrogels (Ø10 mm×10 mm) were performed (1 mm/min, 60% strain). The recoverability of the hydrogels was tested by performing 5 repetitions of compression–relaxation at a constant compressive strain of 40%. The FTIR analysis was tested using Spectrum Two (Perkin Elmer, USA). The XRD were tested in the same manner as the CeO_2_NPs was measured above. The water absorption properties of Gel/SA/CeO_2_ hydrogels were evaluated by calculating the weight change before and after soaking in PBS (pH = 7.4). First, a known weight (*W*_0_) of hydrogel was immersed in 20 ml of phosphate buffered solution (PBS, pH = 7.4) at room temperature, and then the hydrogel was taken out at regular intervals. After gently absorbing the excess water on the surface with filter paper, the weight of the hydrogels was recorded, denoted as *W*_t_. Three parallel samples were used for the tests. The water absorption was calculated using the following equation:


(1) $$\text{Water}\;\text{absorption}\;(\%)=\frac{W_{t}-W_{0}}{W_{0}}\times 100.$$

### Release curve of the hydrogel

The release curve of CeO_2_ from the Gel/SA/CeO_2_-0.4% hydrogel was measured by Inductively Coupled Plasma Mass Spectrometry (ICP-MS). Specifically, the prepared hydrogel was placed in a 15-ml centrifuge tube filled with 5 ml of PBS, and after NIR irradiation for 5 min, it was placed in a constant temperature shaker at 37°C. After a certain period of time, the release liquid was collected, and the hydrogel was placed in a new centrifuge tube and irradiated by NIR. After the collection was completed, the liquid was diluted 10 times with nitric acid and hydrogen peroxide (volume ratio 8:1), heated in a water bath at 70°C until the CeO_2_ dissolved, and the Ce^3+^ content was measured by ICP.

### Rheological measurement

The dynamic rheological tests of Gel/SA/CeO_2_ hydrogels with different CeO_2_NPs contents were characterized by a rotational rheometer (Discovery DHR-20, TA Instruments, USA). Before performing the temperature sweep test, hydrogel samples (Ø10 mm×1 mm) were placed between 20 mm parallel plates and the periphery was sealed by silicone oil to prevent the evaporation of water. Subsequently, temperature sweep tests were performed at temperature ranging from 0 to 60°C at a constant strain of 0.1%, a constant frequency of 10 rad/s, and a heating rate of 5°C/min to study the temperature responsiveness of the hydrogels. The storage modulus (*G*′) and loss modulus (*G*″) of the hydrogels were tested at a constant strain of 0.1% and a constant temperature of 37°C in the frequency range of 0.1–100 rad/s to evaluate the stiffness of the hydrogels.

### Photothermal performance of the hydrogels

The photothermal properties of the hydrogels were evaluated using a fiber-coupled diode laser system (MD-III-808, Changchun New Industry Optoelectronic Technology Ltd, China). Each group of hydrogel samples (Ø10 mm×5 mm) was placed under 808 nm laser, and irradiated with laser power of 0.5, 1.0, 1.5 and 2.0 W/cm^2^ for 5 min, respectively. During irradiation, the temperature was recorded with an infrared thermal imaging system (Fotric 226S, Feicuke Smart Technology Co., Ltd, China) and infrared pictures were taken every 60 s.

### *In vitro* antibacterial activity

The spread plate count method was used to assess the hydrogel’s *in vitro* antibacterial effectiveness against Gram-negative *E.coli* and Gram-positive *S.aureus*. First, the sterilized hydrogel samples (Ø10 mm×5 mm) were placed in a 24-well plate, and 100 µl of bacterial suspension (1 × 10^5^ CFU/ml) was incubated on the surface of the hydrogels with or without irradiation (808 nm, 1.5 W/cm^2^) for 5 min. Then 900 µl of PBS was used to wash the bacteria solution. The same conditions were used to incubate bacterial suspensions (100 µl) in PBS (900 µl) for control samples. Subsequently, 50 µl of the obtained bacterial suspension from each treatment group was evenly spread on LB agar plate. Colonies were formed after 12 h at humidified incubator containing 5% CO_2_ at 37°C, the colonies on the plates were photographed and counted.

The remaining bacterial suspension samples were centrifuged (1000 rpm, 10 min) to collect the pellet and fixed in 1.5% glutaraldehyde solution overnight. Bacterial samples were dehydrated with gradient ethanol solution (30, 50, 70, 80, 90, 100%), and then dropped onto silicon wafers. After natural volatilization, the morphology of bacteria treated with different group was observed by SEM.

### Cell biocompatibility assessment

The cytotoxicity of hydrogels was evaluated using a direct contact test between hydrogels and cells. First, HUVECs and each group of hydrogel samples were seeded in 96-well plate. After irradiating with near-infrared light (808 nm, 1.5 W/cm^2^) for 5 min, co-cultivate at 37°C in a humidified incubator containing 5% CO_2_. Well plates without added hydrogels were used as a control group, and well plates without cells were named blank. After 1, 3, 5 and 7 days of treatment, the samples and medium were removed and 100 µl of CCK-8 solution was added to each well for 2 h in dark. Then, absorbance at 450 nm was obtained by a microplate reader (Bio-Tek, Hercules, USA). The cell viability (CV) was calculated by Equation (2): where CV is the cell viability, OD_t_ is the mean value of the experimental wells, OD_0_ is the mean value of the blank wells and OD_c_ is the mean value of the control wells.


(2) $$\text{CV}\;(\%)=\frac{{\text{OD}}_{t}-{\text{OD}}_{0}}{{\text{OD}}_{c}-{\text{OD}}_{0}}\times 100,$$

Morphology of live and dead cells was tested by AO/EB staining kit. Similarly, HUVECs and each group of hydrogel samples were seeded in 24-well plate at a density of 1 × 10^4^ cells/well. Continue the incubation after 5 min of NIR (808 nm, 1.5 W/cm^2^) irradiation. Meanwhile, well plates without hydrogel added were used as the control group. After 1, 3, 5, and 7 days of treatment, The hydrogel disks and medium were removed and 500 µl of AO/EB staining solution was added to each well and incubated for 15 min at 37°C without light. The red and green fluorescence of cells were observed under a fluorescence microscope (AXIO Observer 3, Carl Zeiss, USA) and images were taken randomly in the field of view.

### Intracellular ROS scavenging

To determine the antioxidative ability of the hydrogels, H_2_O_2_ was used to induce oxidative stress. Briefly, HUVECs were incubated at 37°C and 5% CO_2_ for 24 h to obtain cell adhesion, and then stimulated with medium containing H_2_O_2_ (100 µM) for 3 h to induce oxidative stress. Next, each group of hydrogel samples was added to the well plate and replaced with fresh medium to continue incubation. After 24 h of incubation, they were fixed in 4% paraformaldehyde and permeabilized with 0.1% Triton X-100 for 5 min. One hundred microliters of freshly prepared iFluorTM^555^ phalloidin working solution were added to the well plate and incubated at room temperature for 90 min in the dark. After washing with PBS, 2′-7′dichlorofluorescin diacetate (DCFH-DA) staining solution was added and incubated at 37°C for 30 min without light. After washing with PBS, 4′,6-diamidino-2-phenylindole (DAPI) was added for counterstaining at room temperature for 5 min in the dark. Fluorescence imaging of cells was observed under a laser confocal microscope (CLSM 880, Carl Zeiss, USA) after washing with PBS. The group received no treatment was named as the negative group, while the group that continued to culture with fresh medium without adding hydrogels after induction with H_2_O_2_ was named as the positive control.

### Cell scratch experiment

The ability of hydrogel to promote cell migration was tested by cell scratch assay. HUVECs cells were seeded in 12-well plate at a density of 5 × 10^4^ cells/well and incubated with complete growth medium containing 10% fetal bovine serum (FBS). A monolayer of cells was formed after 24 h. Use a-200 µl pipette tip to scrape the monolayer of cells, and the scratch area is recorded as *S*_0_. Next, the sterilized hydrogel was placed in the well to contact the scratch, and the medium was changed to medium containing 0.1% FBS to continue the culture. Media containing 0.1% FBS has been reported to inhibit cell proliferation and ensure that *in vitro* wound closure is caused only by cell migration [51]. Each well plate was irradiated with NIR (1.5 W/cm^2^, 5 min) and then placed in the incubator. After incubation for 24, 48, and 72 h, the medium and hydrogels were removed, and the cell migration was photographed with a microscope. The area of the scratched area was recorded as *S_t_*. The healing rate of cell scratches was calculated by the following equation:


(3) $$\text{Cell}\;\text{scratch}\;\text{healing}\;\text{rate}\;(\%)=\frac{S_{0}-S_{t}}{S_{t}}\times 100.$$

### Western-blot analysis

Angiogenesis-related proteins secreted by cells were detected by western immunoblotting (WB). After HUVECs were incubated with each group of hydrogels for 5 days (irradiated with 1.5 W/cm^2^ power of NIR for 5 min), the cells were collected. First, total protein was extracted with radioimmunoprecipitation assay buffer (RIPA buffer, Servicebio, China) and quantified by BCA protein kit. Afterwards, equal amounts of proteins were separated using SDS-polyacrylamide Gel electrophoresis (SDS–PAGE) and transferred onto polyvinylidene fluoride (PVDF, 0.45 µm, Servicebio, China) membranes. Then, the PVDF membranes were blocked with 5% nonfat milk solution for 30 min and incubated with specific primary antibodies overnight at 4°C in a shaker. Finally, the PVDF membrane was incubated with the secondary antibody for 30 min at room temperature. The membranes were visualized with enhanced chemiluminescence (ECL, Servicebio, China) reagent and chemiluminescence imaging system.

### PCR analysis

The expression of cellular angiogenesis-related factors was detected using cellular expression polymerase chain reaction (PCR). Total mRNA was extracted from HUVECs using Trizol reagent according to the manufacturer’s protocol, and then converted to cDNA using the Prime Script™ RT reagent Kit (Takara). Subsequently, PCR was performed using a Light Cycler 480 SYBR Green I Master (Takara). The primer sequences for the genes used in the experiments are listed in Supplementary Table S1. The expression of GAPDH gene was set as the internal control for each sample. The results were analyzed using the $2^{-\Delta \Delta C_{t}}$ method.

### *In vivo* hemostatic activity

Rat liver injury and tail amputation models were used to assess the hydrogels’ hemostatic capabilities. The rats were fixed to a cork board while under anesthesia for the rat liver injury experiment. A pre-weighed filter paper was positioned below the rat liver after the belly was sliced open using surgical scissors to reveal the organ. A scalpel was used to cause hepatic hemorrhage, and several samples (0.05 g CeO_2_NPs, 10-mm diameter Gel/SA and Gel/SA/CeO_2_ hydrogels) were subsequently applied directly to the bleeding area. The control group was that which received no care following a hepatic hemorrhage. Blood loss and hemostasis time were measured and examined.

Similar procedures were performed for the rat tail amputation model. First, cut at 1/3 the length of the tail and place pre-weighed filter paper under the bleeding site. Then, each group of samples was gently placed on the bleeding site, amount of blood loss and time of hemostasis were then recorded. The control group was designated as the group that received no therapy.

### *In vivo* bacteria-infected diabetic wound healing assessment

All animal experiments complying with the National Research Council’s Guide for the Care and Use of Laboratory Animals were reviewed and approved by the Animal Ethics Committee of Jinan University (Approval No. IACUC-20221109-05), China.

Male Sprague–Dawley (SD) rats (6–8 weeks old) were used to construct a bacterial infection model of diabetic full-thickness skin defect. First, a diabetes model was established: after 16 h of fasting, male SD rats were injected with streptozotocin (60 mg/kg) through the abdominal cavity, and blood glucose was measured every 3 days. After 4 weeks, the fasting blood glucose level exceeded 16.6 mM, indicating that the diabetic rat model was successfully constructed. Four full-thickness skin wounds with a diameter of 10 mm were made on the back of the successfully modeled diabetic rats after shaving the back hair. After the wounds were made, 100 µl of 1.0 × 10^6^ CFU/ml *S.aureus* was dropped onto the wound surface with a pipette, and evenly spread with a cotton swab to establish a *S.aureus*-infected diabetic wound model. Subsequently, the rat models were randomly divided into 4 groups for treatment, which were named as control (Tegaderm film, 3M), Gel/SA group, Gel/SA/CeO_2_ group and Gel/SA/CeO_2_+NIR group. The wound area was monitored and photographed on Days 0, 3, 7, 10, and 14 after wound creation to assess wound healing performance. The wound healing rate (%) was calculated as follow equation: where *S*_0_ represents the wound area on Day 0 and *S*_t_ represents the wound area at the specified time point.


(4) $$\text{Wound}\;\text{healing}\;\text{rate}\;(\%)=\frac{S_{0}-S_{t}}{S_{0}}\times 100.$$

### *In vivo* antibacterial analysis

The rats were sacrificed on Day 3, and the infected tissues were collected and placed in a sterile centrifuge tube containing 1 ml of PBS and homogenized. After being diluted 1 × 10^4^ times, pipetted 50 µl of the suspension and spread it on the LB agar plate and incubated for 12 h.

### Immunofluorescent, immunohistochemical, and histological analysis

The rats were sacrificed at the predetermined time (3, 7, 10, 14d), then, the tissues around the wound were removed using surgical scissors and fixed in 4% paraformaldehyde for subsequent immunofluorescence staining, immunohistochemical sectioning and histological analysis. Immunofluorescence staining was performed with dihydroethidium (DHE, a ROS prober), and DAPI to assess oxidative stress in different treatment groups on Day 3. CD86 (M1 phenotype macrophages, red) and CD206 (M2 phenotype macrophages, green) immunofluorescence staining was used to detect the degree of wound inflammation in different treatment groups on Day 3. The distribution of neovascular and smooth muscle cells at the wound site on Day 7 was marked by CD31 and α-SMA, and the cell nuclei were counterstained with DAPI. Wound healing process was assessed by hematoxylin and eosin (H&E) staining, Masson’s trichrome (MT) staining.

### *In vivo* toxicity

After the 14-day treatment, the main organs (heart, liver, spleen, lung, and kidney) of the bacteria-infected diabetic rats were collected for H&E staining. The peripheral blood of diabetic rats was collected for serum biochemical index analysis and blood routine examination.

### Statistical analysis

Statistical analysis was performed using GraphPad Prism 7.0 software. Data are presented as mean±standard deviation of minimum 3 parallel samples (*n* = 3). Statistical differences were determined using one-way ANOVA. A value of *P* < 0.05 was considered statistically significant (* for *P* < 0.05, ** for *P* < 0.01, *** for *P* < 0.001).

## Results and discussion

### Characterization of CeO_2_NPs

**Figure 2.**
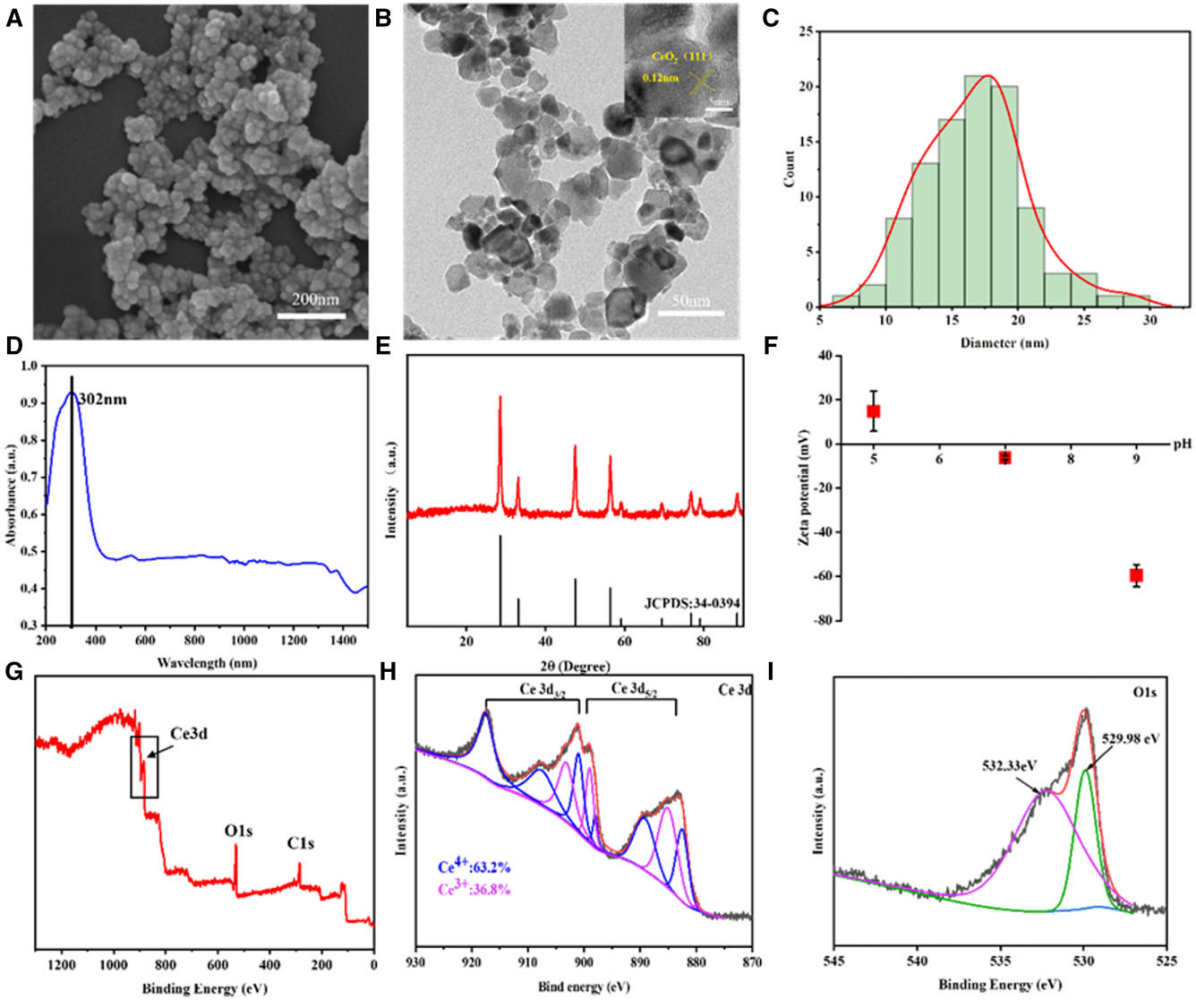
(**A**) SEM images. (**B**) TEM images. (**C**) The particle size distribution chart of CeO_2_NPs. (**D**) UV–vis absorption spectra. (**E**) XRD patterns. (**F**) Zeta potential. XPS spectra of CeO_2_: (**G**) wide, (**H**) Ce 3d and (**I**) O1s.

SEM (Figure 2A), TEM (Figure 2B) and particle size distribution statistics images (Figure 2C) show that the CeO_2_NPs exhibited spherical morphology and have a relatively uniform particle size distribution with an average particle size of 16.27 nm and a lattice spacing of 0.12 nm. The UV-Vis results (Figure 2D) indicate that CeO_2_NPs have characteristic absorption at 302 nm, which is consistent with previous reports [52]. Sharp and intense characteristic diffraction peaks appeared in the XRD pattern of Figure 2E. It showed that the prepared CeO_2_NPs had good crystallinity and a cubic fluorite structure (JCPDS 34-0394). In addition, no other diffraction peaks were observed. This indicates that the obtained samples are of high purity.

Zeta potential results (Figure 2F) show that the values changed from positive (14.8 ± 9.05 mV) to negative (−59.6 ± 5.01 mV) as the PBS buffer solutions changed from acidic (pH = 5) to basic (pH = 9), which is similar to that of previous studies [53], and the reason is due to adsorption of more H^+^ or OH^−^ in acid or basic condition.

XPS (Figure 2G) found that the CeO_2_NPs were mainly composed of C, O, and Ce elements. Figure 2H shows the high-resolution spectrum of Ce, fitting the mixed valence states of Ce^3+^ and Ce^4+^ in the spectrum: the peaks at 880.0, 885.0, 899.0 and 903.1 eV belong to Ce^3+^, while the peaks at 882.5, 889.0, 899.0, 900.9, 907.5 and 917.5 eV peaks represent Ce^4+^. In Figure 2I, the core-level XPS spectrum of O1s is shown, the peak at 529.98 eV is attributed to oxygen vacancies within CeO_2_NPs, while another higher binding energy peak at 532.33 eV is attributed to the surface adsorption of oxygen in H_2_O [54]. Therefore, XPS analysis indicates that both Ce^3+^ and Ce^4+^ are present in the samples, and the percentages of Ce^3+^ and Ce^4+^ were 36.8% and 63.2%, respectively.

### Structure and properties of hydrogels

**Figure 3.**
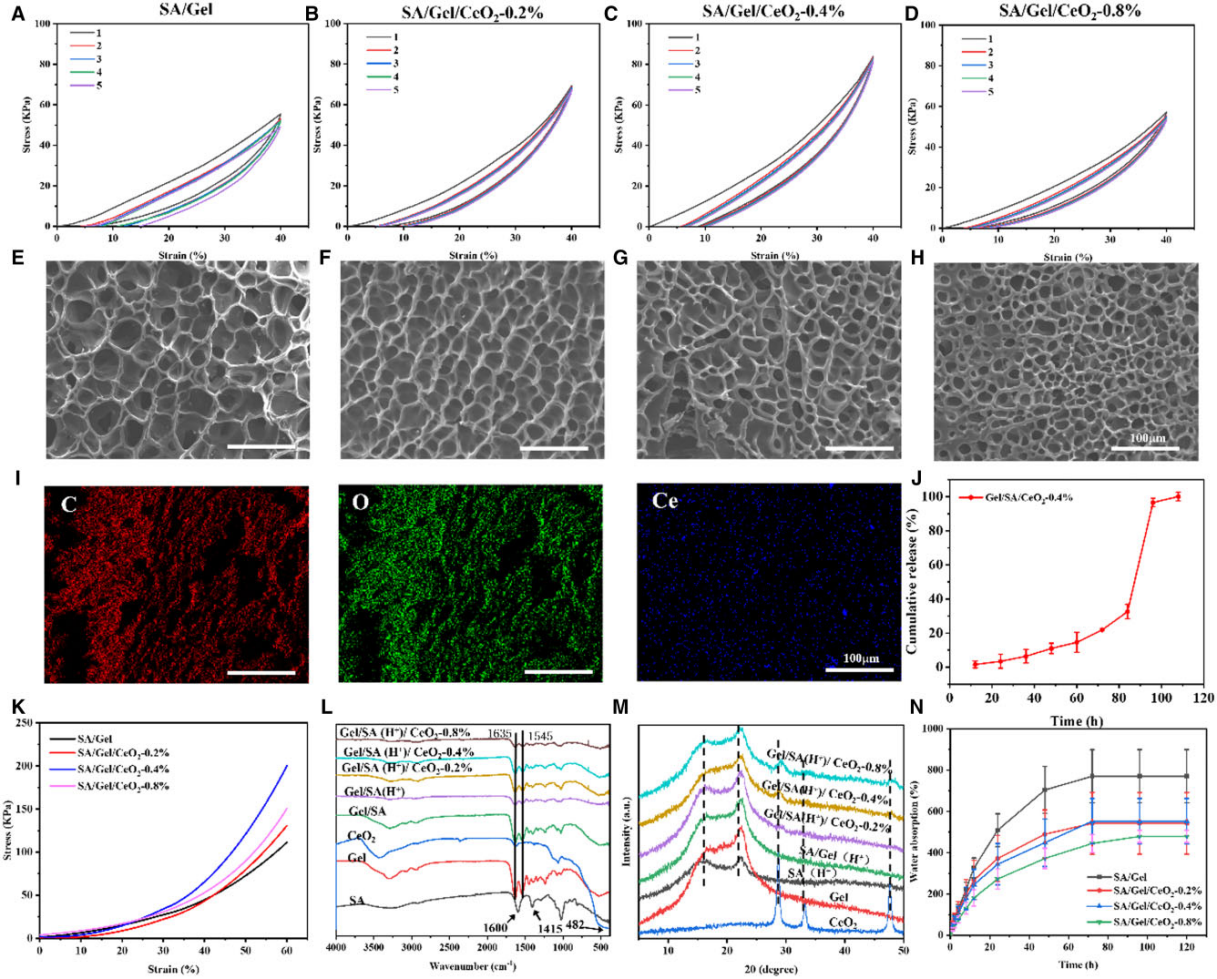
(**A–D**) Cyclic compression curves and (**E**–**H**) SEM images of different hydrogels. (**I**) Mapping images of gel/SA/CeO_2_-0.4% freeze-dried hydrogels. (**J**) Release kinetics curve of the gel/SA/CeO_2_-0.4% hydrogel. (**K**) Stress–strain curves of different hydrogels. (**L**) FTIR spectra of various materials. (**M**) XRD patterns of various materials. (**N**) Water absorption curve of different hydrogels.

Five cyclic compressions results of the Gel/SA/CeO_2_ hydrogels are shown in Figure 3A–D. These hydrogels had a distinct hysteresis loop after the first cycle, and this was caused by initial broken of weak hydrogen bonds and internal friction between polymers [55], resulting in massive energy dissipation. After the first cycle, subsequent stress–strain curves had similar cycle trajectories, indicating good fatigue resistance of the hydrogel. Among them, the hydrogen bonds between the macromolecular chains of Gel, the interaction between Gel and SA, and the crystalline region of SA are the key factors of the excellent mechanical characteristics of hydrogels.

Surface morphology of Gel/SA/CeO_2_ hydrogel was analyzed by SEM. As shown in Figure 3E, the Gel/SA xerogels had many pore structures. With increasing incorporation of CeO_2_NPs, the pore size of the samples became smaller accordingly. This is because CeO_2_NPs play a role as cross-linking points, and the increase of CeO_2_NPs content was equivalent to increasing the cross-linking points of hydrogels, resulting in increased cross-linking density and smaller pore size. In addition, it was observed that Ce element was relatively uniformly dispersed in the hydrogel through the mapping image (Figure 3I).

CeO_2_NPs release curve of Gel/SA/CeO_2_-0.4% composite hydrogel is shown in Figure 3J. The release was slow and steady in 96 h, but increased fast afterwards. This was because CeO_2_NPs generated heat after being irradiated by NIR, and the heat caused the temperature-sensitive Gel network swollen and partly dissolved, resulting a continuous release of CeO_2_NPs. After 96 h, the network collapsed, which caused a large release of CeO_2_NPs. From the above results, we could determine that the hydrogel will be changed every 3–4 days in application.

As shown in Figure 3K, the compressive strength of the hydrogels first increased and then decreased with increasing content of CeO_2_NPs, wherein the compressive stress at 60% deformation was 111.3, 130.8, 200.0 and 150.9 kPa for pure Gel/SA, 0.2%, 0.4%, and 0.8% hydrogel, respectively. This indicates that CeO_2_NPs can enhance the Gel/SA hydrogel to a certain extent, while the higher CeO_2_NPs content (0.8%) reduces the mechanical strength of the hydrogel, may be due to uneven disperse or agglomeration of particles.

FT-IR spectrum of Figure 3L shows that there is just physical crosslinking between Gel and SA, and no chemical reaction occurs. The absorption peaks at 1600 and 1415 cm^−1^ in the infrared spectrum of pure SA were corresponded to the asymmetric and symmetrical stretching vibrations of the –COO– group of alginates, respectively. The absorption peaks of Gel appeared at 1635 and 1545 cm^−1^, which were due to C = O and C–N stretching vibrations (amide I band) and –NH group bending vibration (amide II band). The absorption peak of CeO_2_NPs appeared at 482 cm^−1^, which was due to the stretching vibration of Ce–O [54]. In the infrared spectrum of Gel/SA/CeO_2_ hydrogel, the absorption peaks of CeO_2_NPs and SA overlapped with that of Gel without obvious difference, but the characteristic absorption peaks of each component were retained. This suggests that their structure was not changed after recombination.

Figure 3M shows the XRD pattern of the hydrogel. Gel/SA/CeO_2_ hydrogel contained CeO_2_ diffraction peaks for the (111), (200), and (220) crystal planes, and that the intensities of these peaks increased as the quantity of CeO_2_NPs did. This proves that CeO_2_NPs are effectively incorporated into the Gel/SA matrix.

Figure 3N shows the water absorption properties of the hydrogels. It can be seen that the hydrogels basically reached the water absorption equilibrium after 72 h. And the equilibrium water absorption of pure Gel/SA, 0.2%, 0.4%, and 0.8% hydrogel were 770.3%, 542.1, 552.4, and 478.7%, respectively. The results showed that the water absorption decrease with the addition of CeO_2_NPs. This may be due to the fact that CeO_2_NPs occupy part of the vacancies of the hydrogel network, thereby restricting the expansion of the hydrogel network and hindering the swelling of the hydrogel. In addition, the decrease in pore size is also a cause of the decrease in swelling ability. In addition, it also indicates that CeO_2_NPs enhance the crosslinking density of the hydrogel, which is consistent with the previous mechanical test results. The excellent water absorption is conducive to maintaining a moist environment on the wound surface [56].

### Rheological properties

**Figure 4.**
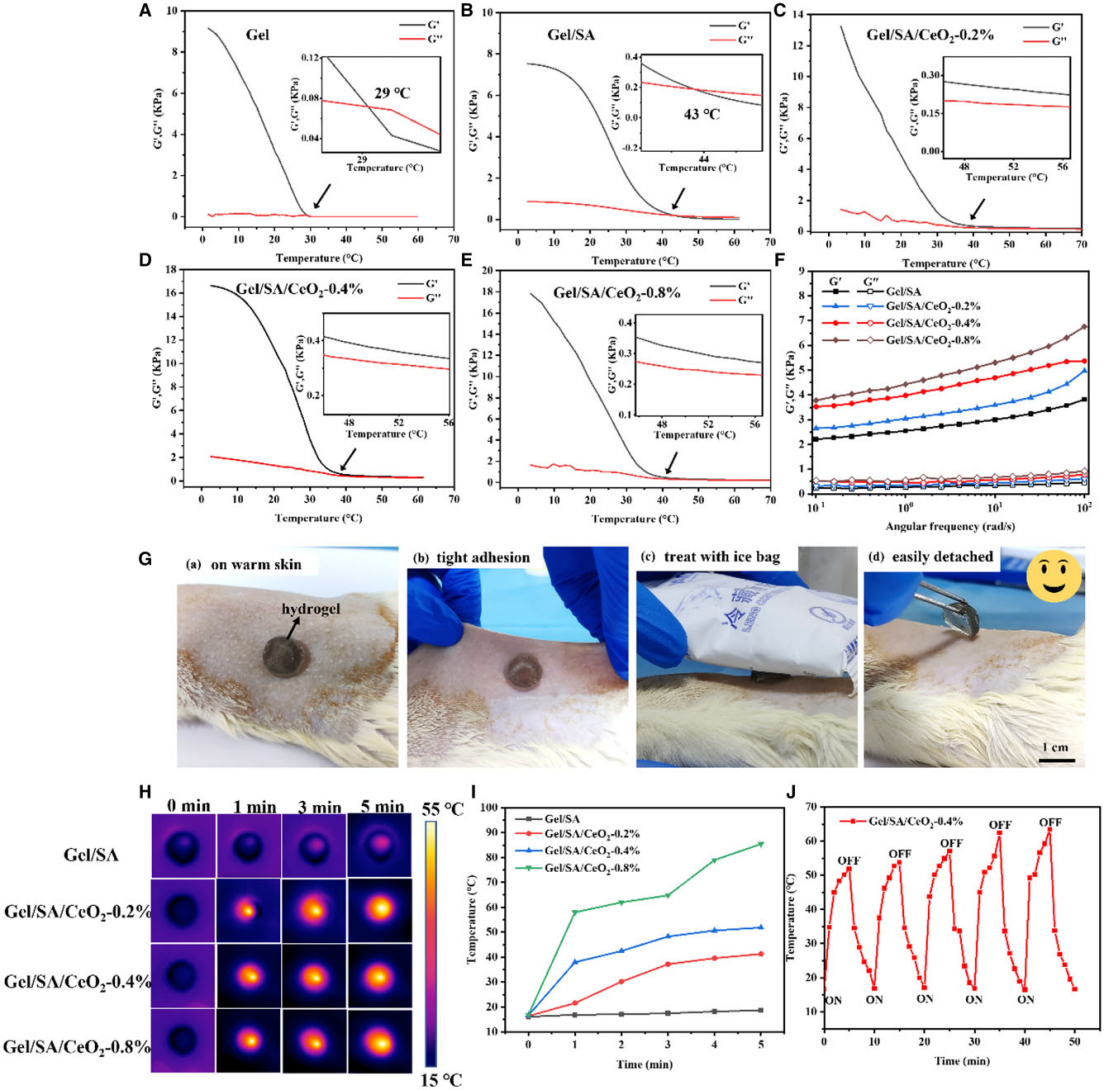
Temperature dependence of the storage modulus (*G*′) and the loss modulus (*G*′′) of (**A**) gel, (**B**) Gel/SA (**C**) Gel/SA/CeO_2_-0.2%, (**D**) Gel/SA/CeO_2_-0.4% and (**E**) Gel/SA/CeO_2_-0.8%. (**F**) Rheological measurement of oscillation angular frequency test for hydrogels. (**G**) Photographs of adhesion and triggerable detached of the gel/SA/CeO_2_-0.4% on rat skin surface. (**H**) Thermal infrared images of hydrogels under NIR irradiation. (**I**) Temperature change curves of hydrogels at a series of concentrations of CeO_2_NPs versus laser irradiation time. (**J**) Temperature rising curves of gel/SA/CeO_2_-0.4% hydrogel during five laser irradiation on/off cycles. An 808-nm laser with the power density of 1.5 W/cm^2^ was used.

The thermal stability of the hydrogels was investigated by oscillatory temperature sweeps. The upper critical solution temperature (UCST) of pure Gel was 27°C (Figure 4A), and the UCST of Gel-SA was increased to 43°C (Figure 4B), which is because the crystalline domains of alginic acid act as physical cross-linkers to the SA network. It is worth noting that the Gel/SA/CeO_2_-0.2%, Gel/SA/CeO_2_-0.4%, Gel/SA/CeO_2_-0.8% did not show UCST even when the temperature was up to 55°C (Figure 4C). This is because CeO_2_NPs acts as cross-linking points, which is beneficial to the entanglement of macromolecular chains and increases the cross-linking density. The above results show that the prepared hydrogel has good thermal stability, which overcomes the disadvantage of poor thermal stability of traditional Gel. More importantly, it is shown that the hydrogel can maintain a good gel state while releasing cerium oxide, instead of becoming a liquid loss, providing a basis for its temperature-responsive release. The angular frequency sweep test results of the hydrogel (Figure 4F) showed that the hydrogel’s loss modulus (*G*′) and storage modulus (*G*′) steadily rose with the addition of CeO_2_NPs. And the *G*′ of the hydrogel was always higher than the *G*″ value, indicating that all samples still maintained the solid hydrogel morphology. This indicates that the Gel/SA/CeO_2_-X hydrogel has good strength and toughness and has the potential to be applied as wound dressings.

Due to the temperature-responsive ability of Gel, the structure of the hydrogel network can be changed at different temperatures. Taking advantage of this, the temperature-responsive adhesion of the composite hydrogel to skin tissue was investigated. After placing the Gel/SA/CeO_2_-0.4% hydrogel on the skin of the dehaired rat and pressing the rat for about 10 s, the hydrogel could adhere to the skin tissue and adapt to the deformation of the skin. An ice pack was then applied to trigger separation of the hydrogel without pulling on the skin (Figure 4G). However, the use of commercial medical tape caused skin pulling, bringing secondary injury to the wound (Supplementary Figure S1). This is because multiple interfacial bonds are formed between reactive groups (such as amino, hydroxyl, and carboxyl groups) on the hydrogel surface and amine or thiol groups on the warm tissue surface immediately after contact with the skin [57]. At low temperature, a large number of hydrogen bonds are formed inside the hydrogel, which is even stronger than the force between the hydrogel and the skin surface. At this point, the hydrogel can be successfully peeled off the skin without damage.

### Photothermal effect of hydrogels

Photothermal effect of the Gel/SA/CeO_2_-X was evaluated under near-infrared (808 nm, 1.5 W/cm^2^) irradiation for 5 min. The real-time infrared thermal image and heating curve distribution of the hydrogel were shown in Figure 4H and I. The video of Gel/SA/CeO_2_-0.4% hydrogel under NIR irradiation was in Supplementary Data. It could be seen that the temperature of Gel/SA hydrogel had almost no change, while the hydrogel containing CeO_2_NPs demonstrated good photothermal conversion performance with the rapid increase of temperature on surface. The light-to-heat conversion mechanism of CeO_2_ may be that under the irradiation of light with an energy equal to or higher than the band gap of CeO_2_, electrons transition from the valence band to the conduction band, while holes are generated in the valence band to form electron-hole pairs. When the excited electrons return to the ground state and recombine with holes, it will cause local heating of the lattice, resulting in a photothermal effect (Supplementary Figure S9) [58, 59].

The temperature change curve of the hydrogel under five cycles of laser irradiation is shown in Figure 4J. The temperature peak of the hydrogel gradually increased during cycling, maybe due to increase of thermal conductivity caused by water evaporation. These tests prove that the composite hydrogel has good photothermal stability and reproducibility. Besides, the photothermal effect of Gel/SA/CeO_2_-X is positively correlated with the NIR radiation power (Supplementary Figure S2).

### *In vitro* antibacterial performance

**Figure 5.**
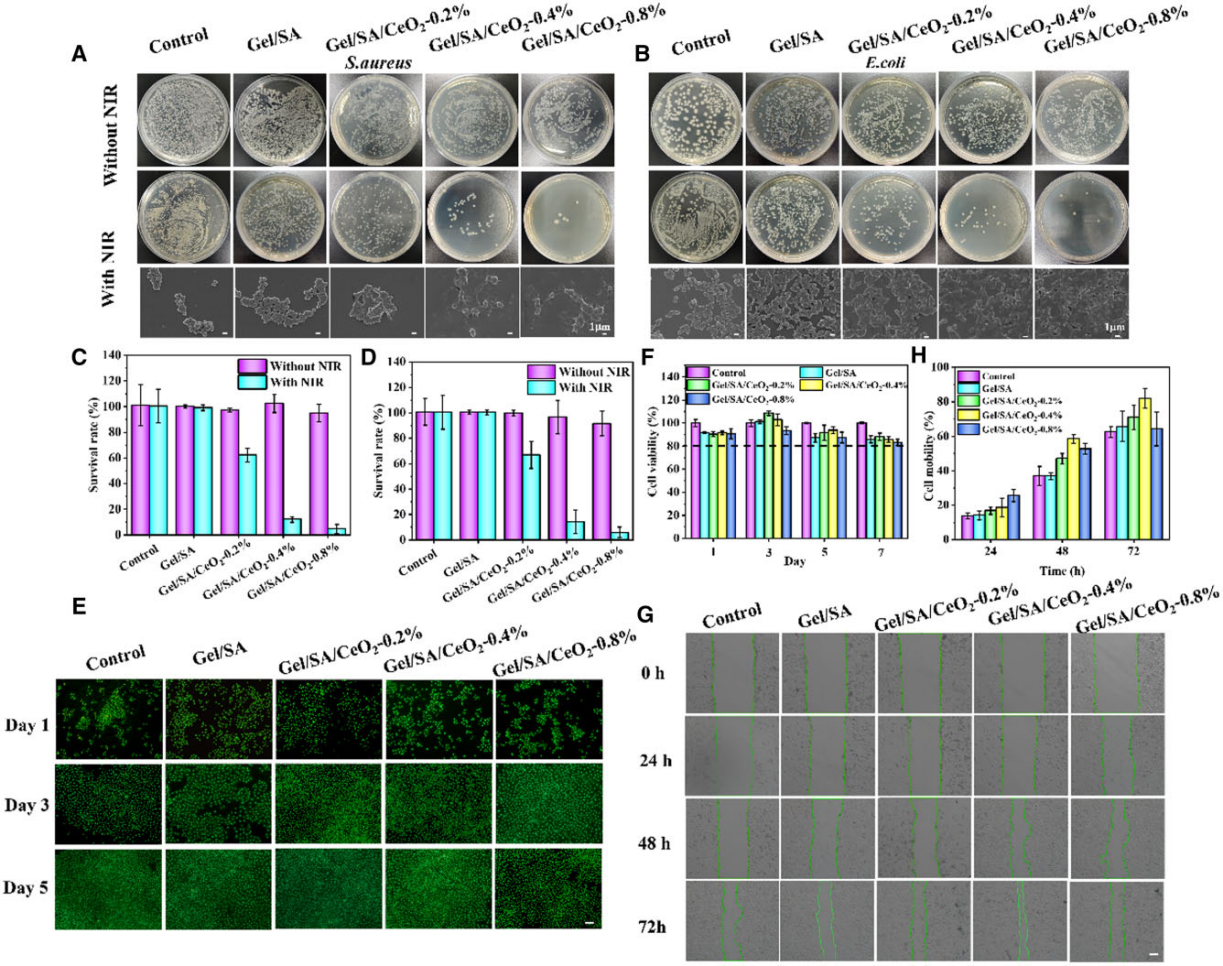
(**A**) Photographs of the bacterial colonies of *S.aureus* and (**B**) *E.coli* and the corresponding SEM images in different condition (scale bar = 1 μm). (**C**) Relative bacterial viabilities by statistical analysis of colonies of *S.aureus* and (**D**) *E.coli*. (**E**) Live/dead staining of HUVECs after being treated with hydrogels for 1, 3 and 5 days (scale bar = 100 μm). (**F**) Cell viability of HUVECs treated with hydrogels for 1, 3, 5 and 7 days. (**G**) Photographs of cell migration in different hydrogels (scale bar = 100 µm). (**H**) Quantitative assessment of cell migration by image J.

The antimicrobial activity of the hydrogels was investigated using *E.coli* and *S.aureus* as experimental models. As shown in Figure 5A and B, it was found that no obvious antibacterial properties were observed in the control group or the Gel/SA hydrogel group, regardless of whether NIR irradiation was performed or not. This phenomenon indicates that Gel/SA itself had no antibacterial effect, and NIR irradiation had no effect on bacterial growth. Similarly, no antibacterial effect could be observed when the Gel/SA/CeO_2_-X hydrogel without NIR, mainly because CeO_2_NPs are trapped in the hydrogel network and cannot be released to contact bacteria within 5 min, therefore the sterilization effect cannot be achieved. However, after NIR treatment for 5 min, the number of colonies in the Gel/SA/CeO_2_-X hydrogel-treated group gradually decreased. Besides, with the increased of CeO_2_NPs content, the antibacterial effect was enhanced. This is because CeO_2_NPs convert the absorbed NIR into thermal energy due to the photothermal conversion ability, and high temperature causes bacterial cell membrane destruction and protein denaturation [60], resulting in bacterial death. At the same time, due to the temperature-responsive properties of the hydrogel, the macromolecular chains unwind after being locally heated to release the CeO_2_NPs from the hydrogel network, and the CeO_2_NPs can exert their antibacterial properties when they are exposed to bacteria. Under the two synergistic antibacterial effects, the Gel/SA/CeO_2_-0.8% hydrogel group could kill more than 95% of bacteria (Figure 5C and D, bacterial survival rate, 3.5% of *S.aureus* and 4.2% of *E.coli*).

### Cell biocompatibility of hydrogels

The cytotoxicity was assessed by co-incubating HUVECs with the hydrogel for a certain period of time. After treatment, AO/EB staining was used to observe cytotoxicity (Figure 5E). It was observed that most of the HUVECs in each group all displayed normal morphology. In addition, cell viability was detected using CCK-8 kit (Figure 5F). Results showed that after 1, 3, 5 and 7 days of co-incubation, the cell viability in each experimental group was higher than 80%.

### *In vitro* cell migration

Migration of endothelial cells is one of the requirements for neovascularization. The ability to promote cell migration was assessed by fabricating scratches in monolayers of HUVECs and then co-incubating with the hydrogels for a certain time. As shown in Figure 5G and H, cells showed different degrees of migration after treatment with different samples. The semi-quantitative analysis of the scratch area showed that the cell migration rate in the Gel/SA/CeO_2_-0.4% group (81.9%) was significantly higher than that in the control (62.5%), Gel/SA (65.6%) and Gel/SA/CeO_2_-0.2% (70.9%) group after 72 h. This indicates that the addition of CeO_2_NPs can effectively promote cell migration. It has also been reported that CeO_2_NPs can promote cell migration [6], which is consistent with this result. However, the cell migration rate (64.3%) of the Gel/SA/CeO_2_-0.8% group was a little lower than that of Gel/SA (65.6%), indicating that the suitable addition of CeO_2_NPs would benefit cell migration. The cytotoxicity of CeO_2_NPs to HUVECs is shown in Supplementary Figure S3. It could be seen that when the concentration of CeO_2_NPs was in the range of 0–1000 µg/ml, the cell viability was greater than 90%, and most of the cells were in good shape. However, the cell viability of 2000 µg/ml CeO_2_NPs was well below than 80%, indicating that CeO_2_NPs showed cytotoxicity when the concentration exceeded a certain value.

### ROS scavenging capacity

**Figure 6.**
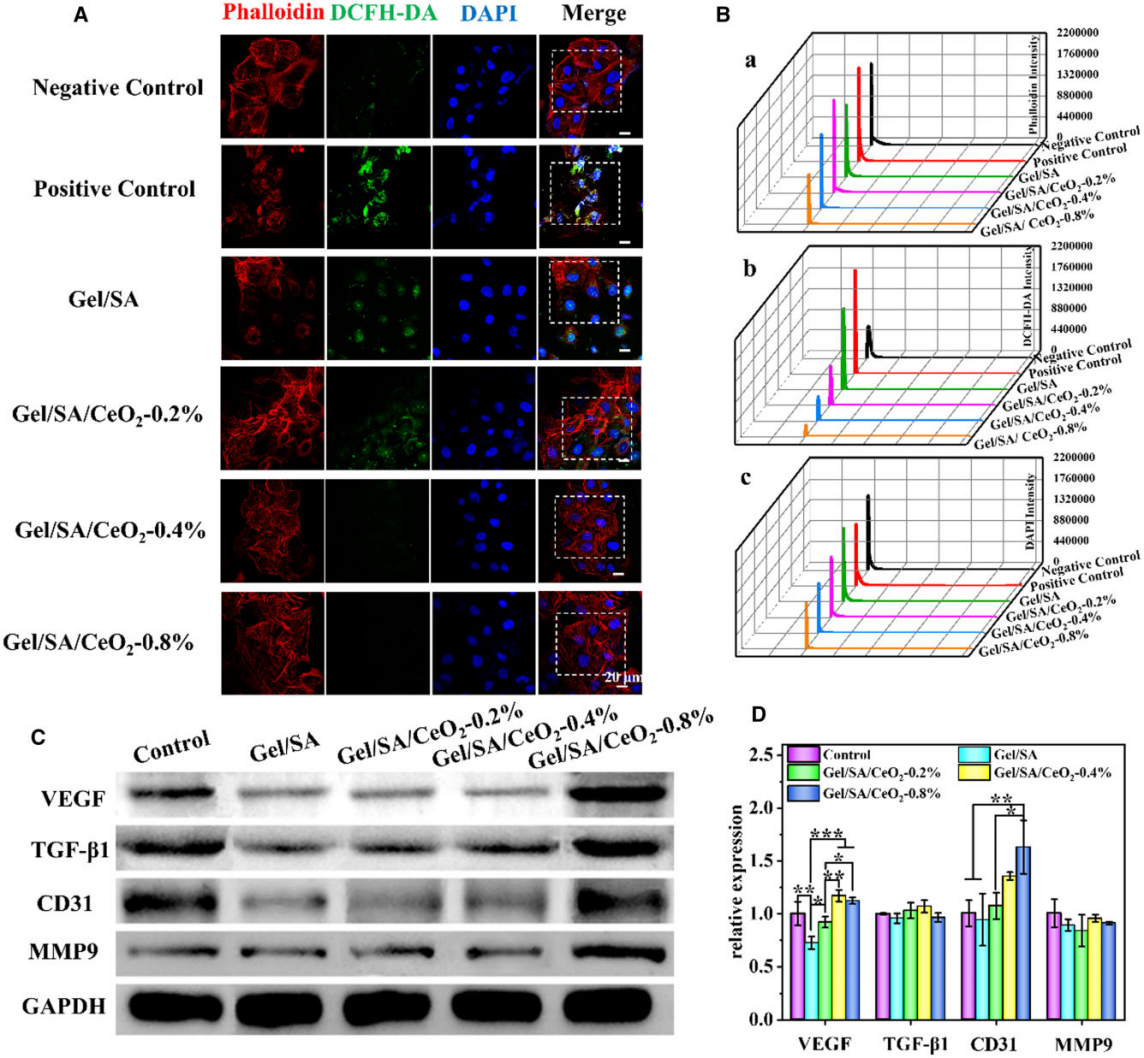
(**A**) Immunostaining of phalloidin (red), DCFH-DA (green) and DAPI (blue) in HUVECs by confocal laser microscopy (scale bar = 20 μm). (**B**) Quantified fluorescence intensity from (**A**). (**C**) Expression of VEGF, TGF-β1, CD31 and MMP9 in HUVECs by western blotting on Day 5. (**D**) Gene expression of VEGF, TGF-β1, CD31 and MMP9 in HUVECs analyzed by PCR on Day 5.

Excessive ROS in wounds leads to prolonged inflammatory responses, making wounds difficult to heal. Studies have shown that H_2_O_2_ is the most stable and abundant ROS in the body [61], so reducing H_2_O_2_ levels is essential to promote wound healing. The ability of CeO_2_ to scavenge H_2_O_2_*in vitro* was evaluated by a H_2_O_2_ assay kit, and the result were shown in Supplementary Figure S4. In general, the H_2_O_2_ clearance rate and CeO_2_NPs concentration showed a dose-dependent relationship, and 2 mg/ml CeO_2_NPs had a good H_2_O_2_ clearance rate (over 80%) after co-incubation for 24 h. Subsequently, the intracellular ROS scavenging rate of the composite hydrogel was evaluated by the ROS indicator DCFH-DA. As shown in Figure 6A, in the positive control group, a strong green fluorescence (ROS) signal could be seen around the blue fluorescence (nucleus), indicating that a large amount of ROS was generated in the cells due to stimulation by H_2_O_2_. Meanwhile, a significant down-regulation of green fluorescence intensity was observed in the Gel/SA/CeO_2_-X-treated group (Figure 6B). Quantitative fluorescence intensity showed that, compared with the positive control group, the ROS fluorescence intensity of Gel/SA/CeO_2_-0.2%, Gel/SA/CeO_2_-0.4% and Gel/SA/CeO_2_-0.8% decreased by 62.8%, 76.7%, and 89.2%, respectively. And the Gel/SA/CeO_2_-0.4% and Gel/SA/CeO_2_-0.8% groups were even close to the negative control group. In addition, by cytoskeleton staining (red), it could be observed that the cells were in a poor state of shrinkage after H_2_O_2_ stimulation, while cells were in a spreading state and grow well after incubation with Gel/SA/CeO_2_-X. It shows that the hydrogel platform has excellent antioxidant capacity, which can effectively remove intracellular ROS and reduce it to normal levels, thereby eliminating oxidative stress, shortening inflammatory stage, and ultimately accelerating wound healing.

### WB analysis and PCR analysis

The proangiogenic capacity of the hydrogels was evaluated by WB and PCR assays. The expression of angiogenesis-related factors was detected by PCR, as shown in Figure 6D. Compared with the blank group, platelet-endothelial cell adhesion molecule (CD31) was significantly expressed in HUVECs incubated with Gel/SA/CeO_2_-X hydrogel. CD31 are frequently used as markers for evaluating angiogenesis [62]. The expression of angiogenesis-related genes usually leads to the expression of related proteins. Therefore, the expression of related proteins was detected by WB. As shown in Figure 6C, after Gel/SA/CeO_2_-X hydrogel treatment, the VEGF, TGF-β1, CD31 and MMP9 genes secreted by HUVECs all effectively expressed related proteins. Compared with the control group, the corresponding protein bands in the composite hydrogel group were more obvious, which also confirmed the pro-vascular properties of Gel/SA/CeO_2_-X. This is mainly due to the excellent pro-angiogenic ability of CeO_2_NPs, the results of which are similar to previous studies [48].

### *In vivo* hemostatic activity

**Figure 7.**
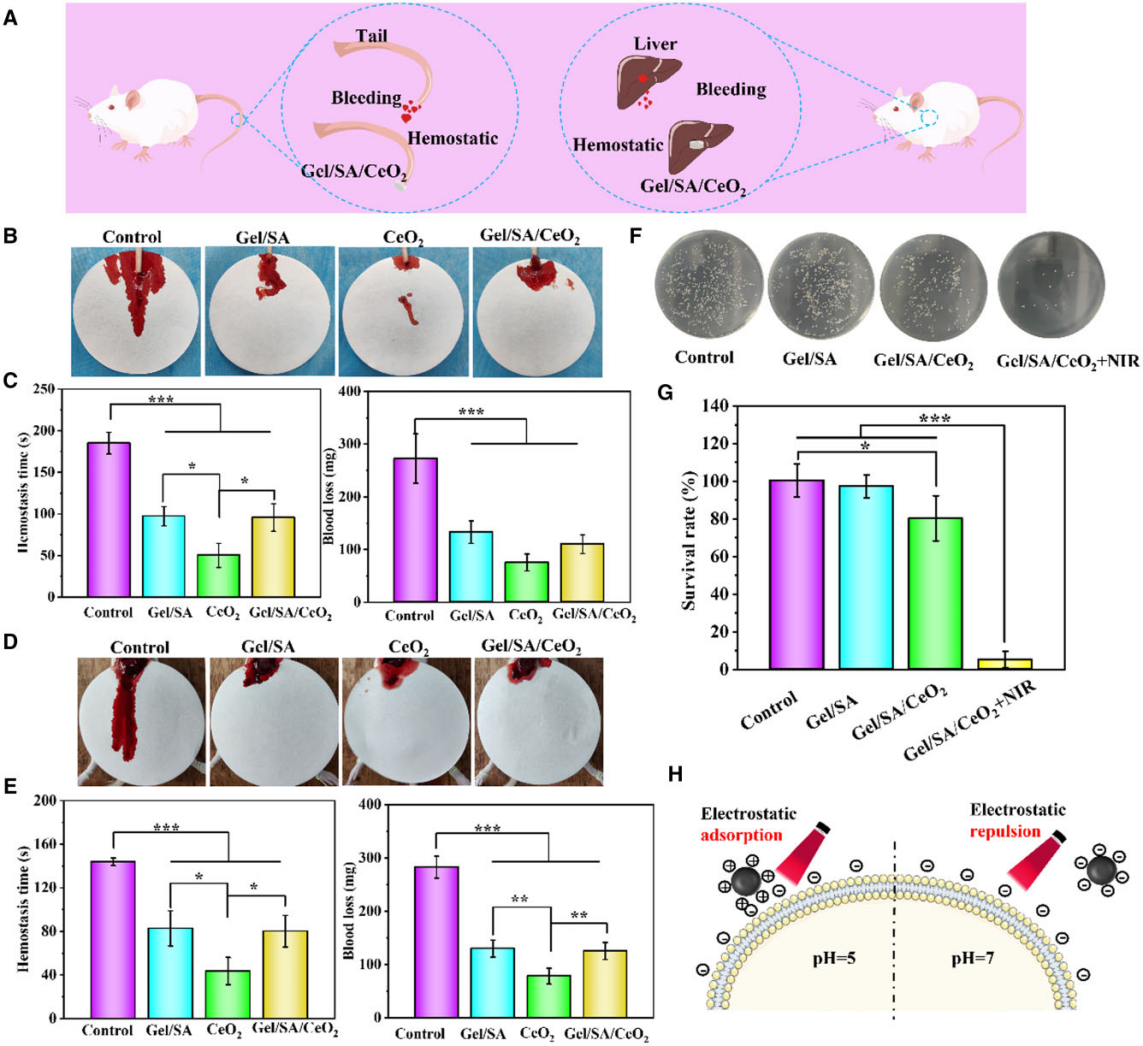
(**A**) Schematic diagram of the rat tail and liver bleeding model and hydrogel hemostasis process. (**B**) Bloodstain photographs of rat tail amputation model and (**D**) rat liver hemorrhage model. (**C**) Quantitative results of blood loss and hemostasis time of rat-tail amputation model and (**E**) rat liver hemorrhage model. (**F**) Representative photographs of bacteria derived from wound tissue in all groups on Day 3. (**G**) Quantitative results of bacteria in wound tissue on Day 3. (**H**) Interaction between positively and negatively charged CeO_2_NPs and a negatively charged bacteria membrane at different pH value.

Taking the photothermal properties and other properties of the hydrogel into accounts, Gel/SA/CeO_2_-0.4% hydrogel was selected for subsequent animal experiments. As the first stage of wound healing, hemostasis is one of the characteristics required for an ideal wound dressing. The hemostatic properties of the Gel/SA/CeO_2_ were evaluated by rat liver hemorrhage model and rat tail docking model, and the operation diagram is shown in Figure 7A. In the rat tail docking model, the amount of tail injury blood loss in the material treatment group was significantly less than that in the control group (Figure 7B). It is evident that all material-treated groups had a reduction in blood loss and a shortening of the hemostasis period (Figure 7C). Among them, the CeO_2_NPs group was the most obvious, and the effects of Gel/SA and Gel/SA/CeO_2_ were comparable. It fully showed that CeO_2_NPs had better hemostatic properties. Likewise, in rat liver model, similar results were obtained. The blood volume and hemostasis time of liver wounds in all material-treated groups were significantly less than those in the control group, while the hemostatic effect of CeO_2_NPs was better than that of pure Gel/SA and Gel/SA/CeO_2_ groups (Figure 7D and E). These results proved the hemostatic effect of Gel/SA/CeO_2_. CeO_2_NPs exhibit high hemostatic properties because CeO_2_NPs are negatively charged in a neutral environment (as shown in Figure 2F), and the contact activation of coagulation factors at the particle-plasma interface is increased, resulting in a higher coagulation velocity [63]. As for Gel/SA/CeO_2_ hydrogels, CeO_2_NPs are trapped in the polymer chains and are not easily released, so Gel mainly provides hemostatic properties, resulting in the hemostatic effect of Gel/SA and Gel/SA/CeO_2_ groups resemblance. Therefore, the hemostatic effect of Gel/SA/CeO_2_ is mainly due to good tissue adhesion ability of Gel to closely fit the wound site, meanwhile, the stable gel network provides a physical barrier to accelerate blood-coagulation, thereby achieving good hemostasis effect.

### Bacteria-infected diabetic wound healing of the hydrogels

Given the excellent antibacterial effect of Gel/SA/CeO_2_-X hydrogel *in vitro*, we collected wound tissue on Day 3 to evaluate *in vivo* antibacterial effect of the hydrogel. As shown in Figure 7F, compared with the control group, the bacterial survival rates of the Gel/SA group and the Gel/SA/CeO_2_ group were almost unchanged, while the bacterial survival rates of the Gel/SA/CeO_2_+NIR group significantly reduced (5.3%), showing excellent antibacterial effect (Figure 7G). Based on the above healing results, it could be concluded that the synergistic treatment combined with CeO_2_ NPs and NIR laser irradiation could effectively eliminate bacteria and promote the wound healing process. CeO_2_NPs exhibited excellent antibacterial properties, as the bacteria produced lactic acid and acetic acid during the growth and metabolism of infected wounds, resulting in local acidification (pH = 4.5 ∼ 6.5) [64]. CeO_2_NPs are positively charged under acidic conditions, and are easily adsorbed to the negatively charged bacterial surface, playing a stronger antibacterial effect (schematic diagram shown in Figure 7H). Furthermore, the antibacterial effect of CeO_2_NPs under different conditions also confirmed this conclusion (Supplementary Figure S5).

**Figure 8.**
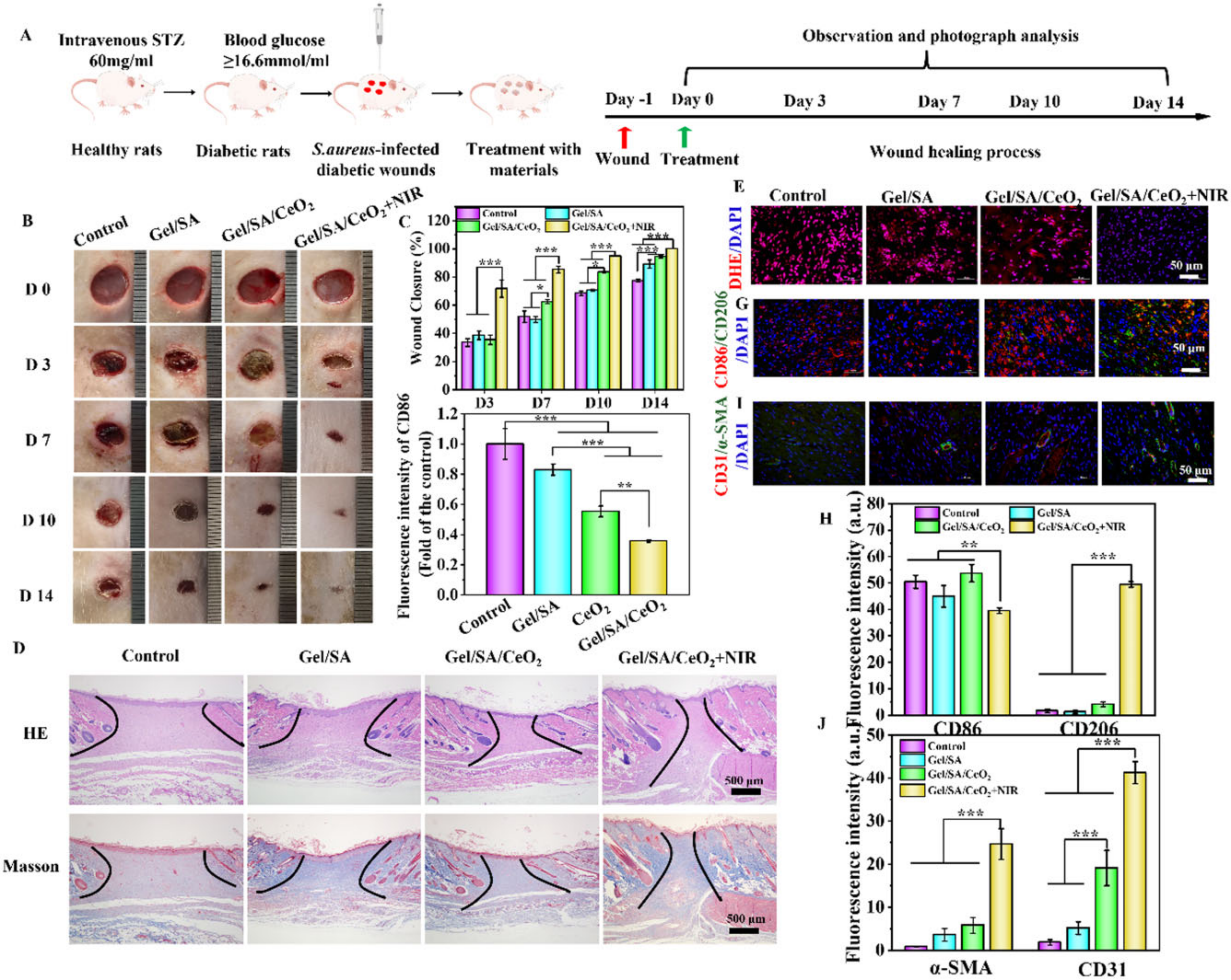
(**A**) Workflow for the assessment of bacteria-infected diabetic wound healing, and schematic diagram of hydrogel treatment. (**B**) Representative photographs of the wound healing process in different groups. (**C**) Quantitative results of the wound closure area at different time intervals. (**D**) Representative H&E staining images of wounds in all groups on Day 14. DHE staining (**E**) fluorescence images and (**F**) quantitative analysis of wounds in each group on Day 3. (**G**) Representative images and (**H**) quantitative analysis of immunofluorescent staining of CD86 (red) and CD206 (green) in each wound tissue on Day 3. (**I**) Representative images and (**J**) quantitative analysis of α-SMA (green) and CD31 (red) immunofluorescent staining of wounds in each group on Day 7. Cell nuclei were stained with DAPI (blue).

The workflow of bacterial infection diabetic wound healing assessment and the schematic diagram of hydrogel treatment are shown in Figure 8A. The wound areas of the different groups were photographed and recorded at specific time intervals, as shown in Figure 8B. On Day 3, all treatment groups showed smaller wound areas. In contrast, the wound closure efficiency of the Control group and the Gel/SA group was poor, accompanied by red and yellow pus exudation. On Day 7, the Gel/SA/CeO_2_+NIR group showed more obvious shrinkage compared with other groups. After 14 days of treatment, the wound area of Gel/SA/CeO_2_+NIR group had been covered with hair and recovered best, while open wounds can still be seen in other groups. Additionally, based on representative images, wound healing traces were created, and each time point’s wound closure rate was then calculated. As shown in Figure 8C, the wound healing rates of the Gel/SA/CeO_2_+NIR group on Days 3, 7, 10 and 14 were 71.7%, 85.4%, 94.9% and 100%, respectively, which were far superior to those of the Control group (33.5%, 51.9%, 68.5% and 77.4%), Gel/SA group (38.7%, 49.7%, 70.6% and 89.2%), and Gel/SA/CeO_2_ group (35.6%, 62.5%, 83.6% and 94.4%). These results indicated that the Gel/SA/CeO_2_+NIR treatment group had a faster healing rate than the other groups. This is because, on one hand, Gel/SA/CeO_2_ hydrogel has excellent photothermal antibacterial ability, which can kill bacteria in time and reduce inflammation (the temperature of the hydrogel treatment was around 50°C, Supplementary Figure S6). On the other hand, CeO_2_NPs can strongly decompose endogenous H_2_O_2_ into O_2_ [65]. Studies have shown that oxygen can effectively improve the survival rate of keratinocytes, fibroblasts and endothelial cells under diabetic hypoxic conditions [66], thereby effectively promoting wound closure.

The healing degree of regenerated skin tissue was evaluated histologically by H&E and MT staining. As shown in Figure 8D, it was observed by H&E staining that granulation tissue was formed in all groups after 14 days of treatment, but the gap of granulation tissue in the Gel/SA/CeO_2_+NIR group was significantly smaller than that in the other groups. Similarly, it was observed from MT staining that the Gel/SA/CeO_2_+NIR group had more collagen deposition than the other groups. Therefore, H&E and MT staining indicated that Gel/SA/CeO_2_+NIR could accelerate wound healing by promoting the formation of granulation tissue and collagen, thereby reaching a satisfactory healing state earlier [67].

DHE staining was used to study the ability of hydrogels to resist oxidative stress damage. The ROS levels observed in Gel/SA/CeO_2_+NIR-treated wounds were significantly lower than those of other treatment groups (Figure 8E and F), indicating that Gel/SA/CeO_2_ hydrogels could effectively scavenge excess ROS during wound healing under NIR irradiation. The degree of inflammation in the wounds of different groups was detected by CD86 and CD206 staining. As shown in Figure 8G and H, compared with other groups, the number of CD206^+^ cells in the Gel/SA/CeO_2_+NIR treatment group was more than that of CD86^+^ cells, indicating that the Gel/SA/CeO_2_ hydrogel can effectively polarize macrophages from M1 to M2, thereby alleviating the inflammatory response. Furthermore, neovascularization and smooth muscle cell distribution at the wound site was assessed by CD31 and α-SMA staining. As shown in Figure 8I and J, Gel/SA/CeO_2_+NIR-treated wounds expressed more CD31 and α-SMA compared with other groups, implying that more blood vessels were forming.

The biosafety assessment of the prepared hydrogel was carried out to measure its potential risk in clinical application. As shown in Supplementary Figure S7, the results of H&E staining analysis of major organs (heart, liver, spleen, lung, and kidney) remained normal after treatment with the composite hydrogel. The blood components of the rats in each group had little change and no abnormality, indicating the functions of the liver and kidney were not affected (Supplementary Figure S8). This is because CeO_2_NPs do not aggregate *in vivo* and can be excreted through the feces [68] or renal system [69]. The above results demonstrated the high biocompatibility of the hydrogel and showed the great potential of this strategy in future clinical translation.

## Conclusion

In this study, a series of biocompatible multifunctional Gel/SA/CeO_2_-X hydrogels containing CeO_2_NPs were prepared, which exhibited rapid hemostasis, efficient bactericidal, antioxidative, and angiogenesis-promoting effects. The Gel/SA/CeO_2_-X hydrogel exhibited good mechanical properties, thermal stability and toughness due to the crystalline domains of SA and the incorporation of CeO_2_NPs. The good tissue adhesion ability and hemostatic ability of Gel provided the hydrogel with good hemostatic effect. The unique valence-state switching properties of CeO_2_NPs endowed the hydrogel with the function of rapid ROS scavenging. In addition, CeO_2_NPs could convert endogenous H_2_O_2_ into O_2_ at the same time of valence state conversion, thus effectively supplying O_2_. CeO_2_NPs synergized with NIR endowed the hydrogel with high antibacterial ability, which could effectively ensure the rapid healing of bacteria-infected diabetic wounds.

To our knowledge, this is the first time that a multifunctional thermosensitive hydrogel has been constructed using the photothermal properties of CeO_2_NPs and applied to promote healing of infected diabetic wounds. Collectively, our Gel/SA/CeO_2_-X composite hydrogels demonstrate a versatile strategy to modulate a wide range of complex microenvironments in diabetic wounds. More importantly, this provides a new direction for the wider application of CeO_2_NPs.

## Supplementary Material

rbad072_Supplementary_Data
