# Supplementary material for: Photothermal effective CeO2NPs combined in thermosensitive hydrogels with enhanced antibacterial, antioxidant and vascularization performance to accelerate infected diabetic wound healing
Source: Regen Biomater. 2023 Sep 1;10:rbad072. doi: 10.1093/rb/rbad072 (PMC10503268; doi:10.1093/rb/rbad072)
Supplement: rbad072_Supplementary_Data [file rbad072_supplementary_data.zip › Revised supplement information.docx]

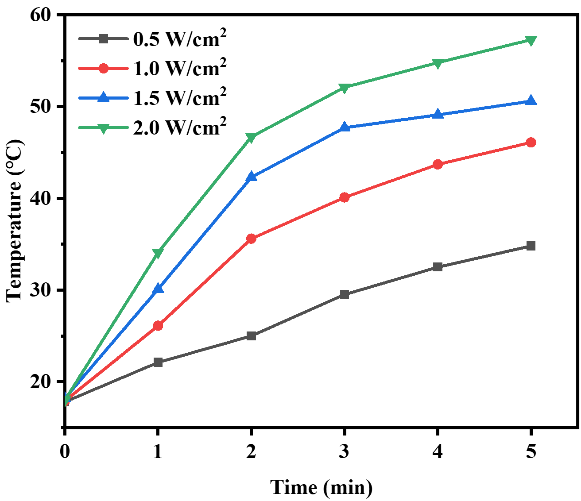

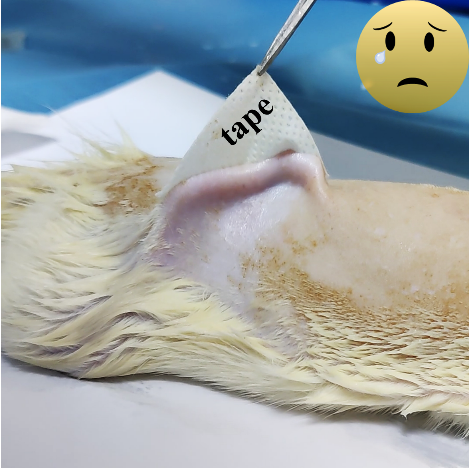
Fig. S1. A photo of medical tape causing skin pulling peeling.


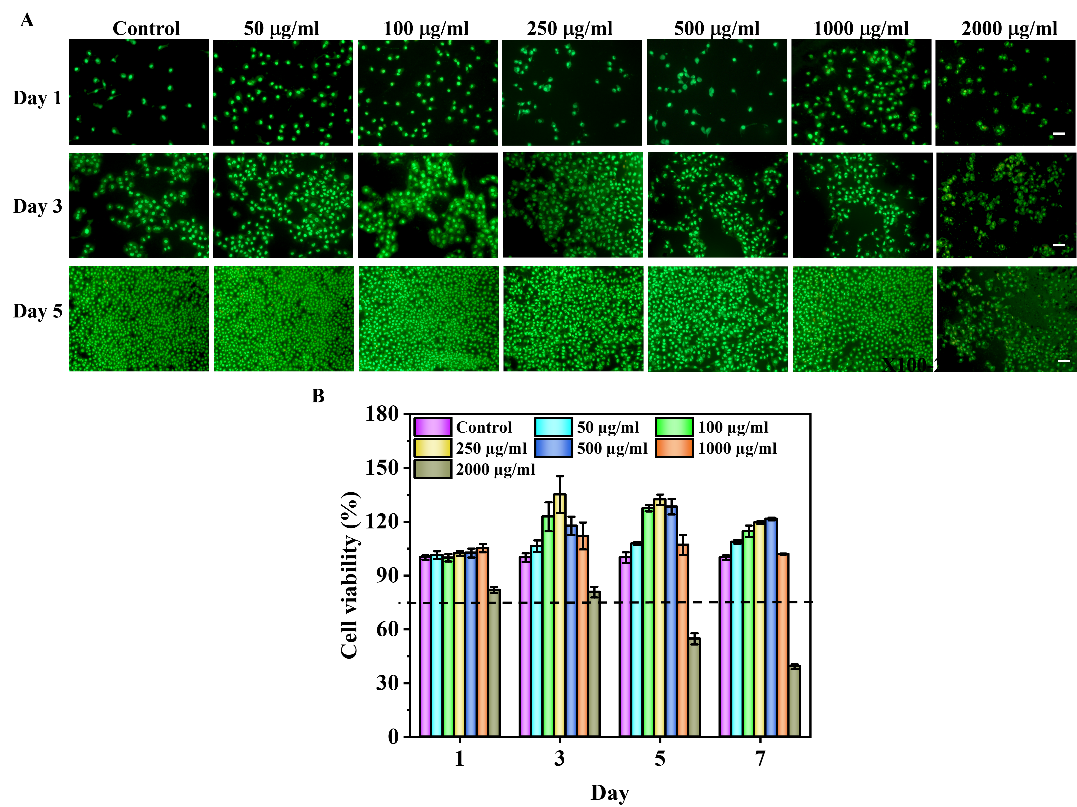
Fig. S2. Temperature curves of Gel/SA/CeO_2_-0.4% upon 808 nm laser irradiation with different powers.

Fig. S3. (A) Live/Dead and (B) Cell viability of HUVECs staining of HUVECs after being treated with different concentrations of CeO_2_NPs for 1,3,5,7 d (scale bar = 100 μm).


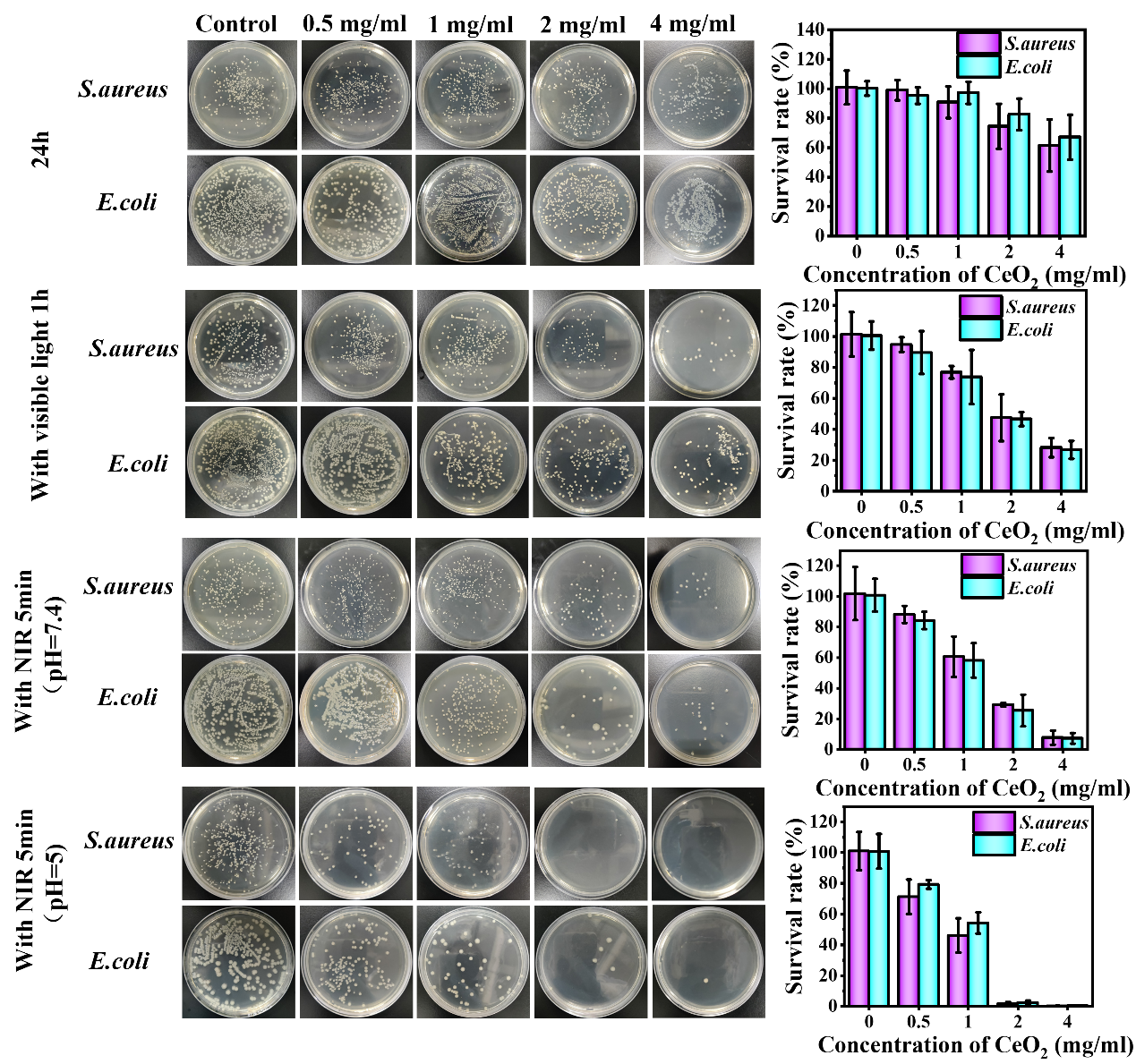

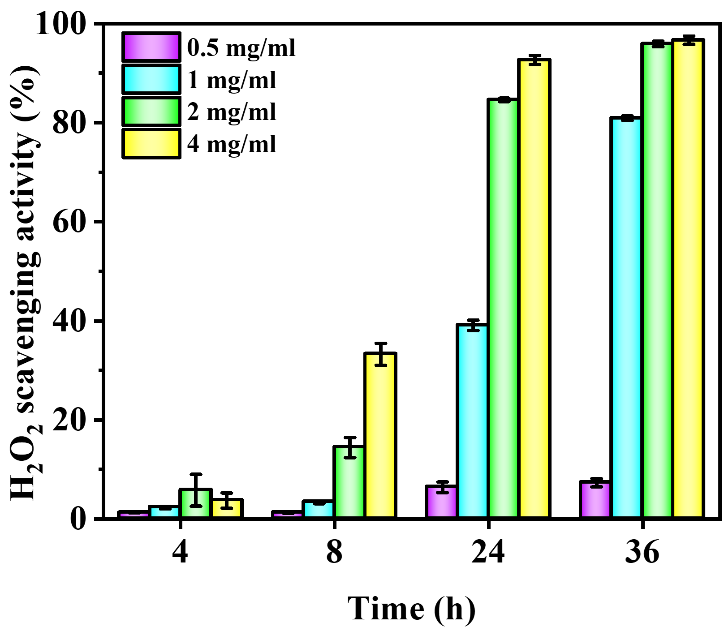
Fig. S4. The ability of CeO_2_NPs to scavenge extracellular H_2_O_2_.

Fig. S5. Antibacterial activities of 0~4 mg/mL of CeO_2_NPs against *E. coli* and *S. aureus* under different condition.

Using *E. coli* and *S. aureus* as experimental models, the antibacterial properties of CeO_2_NPs were evaluated under different conditions. Specifically, the first one is to observe the colony status after co-incubating the CeO_2_NPs suspension with different concentrations directly with the bacterial suspension for 24 hours; In the second condition, mix different concentrations of CeO_2_NPs suspensions with bacterial suspensions and place them under 55W LED light (simulating natural light) for 1 hour to observe the colony status; In the third condition, different concentrations of CeO_2_NPs suspensions were mixed with bacterial suspensions and then irradiated under NIR light and neutral conditions for 5 minutes to observe the colony status; In the fourth condition, different concentrations of CeO_2_NPs suspensions were mixed with bacterial suspensions and irradiated under NIR light and acidic conditions for 5 minutes to observe the colony status. Under the four conditions, the bacterial concentration was 10^4^ CFU/ml.

The result is shown in the figure S5, it could be seen that no matter what the conditions were, the bacteria in the control group grew well (the survival rate is about 100%). The bacterial survival rate after CeO_2_NPs treatment decreased to varying degrees, and with the increase of CeO_2_NPs suspension concentration, the bacterial survival rate decreased, indicating that CeO_2_NPs had a certain effect on antibacterial. Among them, the antibacterial effect was the best after 5 minutes of NIR irradiation in an acidic environment, and almost all the bacteria died (the survival rate of *S. aureus* was only 0.2% ± 0.1, and the survival rate of *E. coli* was only 0.3% ± 0.1). The reason for the large difference in the antibacterial effect of CeO_2_NPs under different conditions is mainly due to the different antibacterial mechanisms.

The antibacterial mechanism of CeO_2_NPs mainly has the following aspects: on the one hand, nanoparticles can be adsorbed on the surface of bacteria, combine with mediators and interfere with cell respiration, DNA replication, cell division, etc. At the same time, the irregular shape or rough and angular surface of nanomaterials may also cause physical damage to bacteria. On the other hand, CeO_2_NPs can produce reactive oxygen species (Reactive Oxygen Species, ROS) species to kill bacteria through a unique mechanism of reversible conversion between Ce^3+^ and Ce^4+^. This bactericidal process is mainly because ROS can chemically degrade various organic components in microorganisms, including DNA, RNA, and proteins, thereby causing great damage to bacteria. Since CeO_2_NPs are positively charged in an acidic environment, they can be better adsorbed to the surface of positively charged bacteria. Combined with multiple mechanisms such as photothermal effect, physical action and ROS
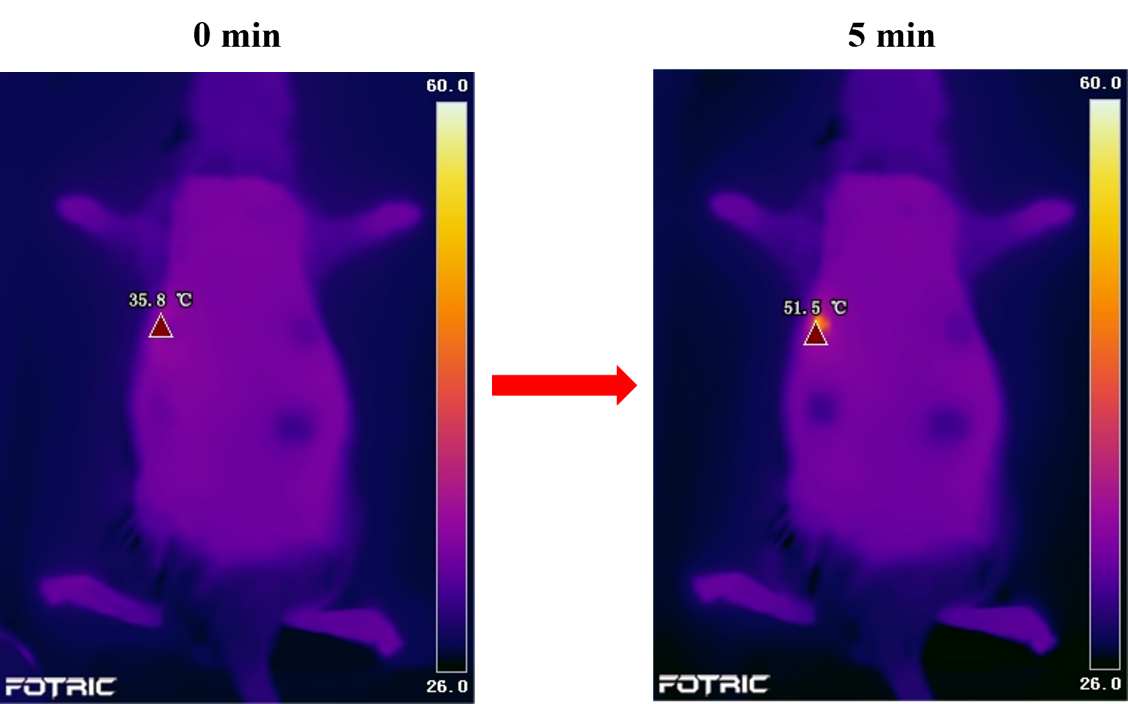
sterilization, it has excellent antibacterial effect (the sterilization rate is close to 100%).

Fig. S6. Thermal image of the composite hydrogel after infrared laser irradiation for 5 min.


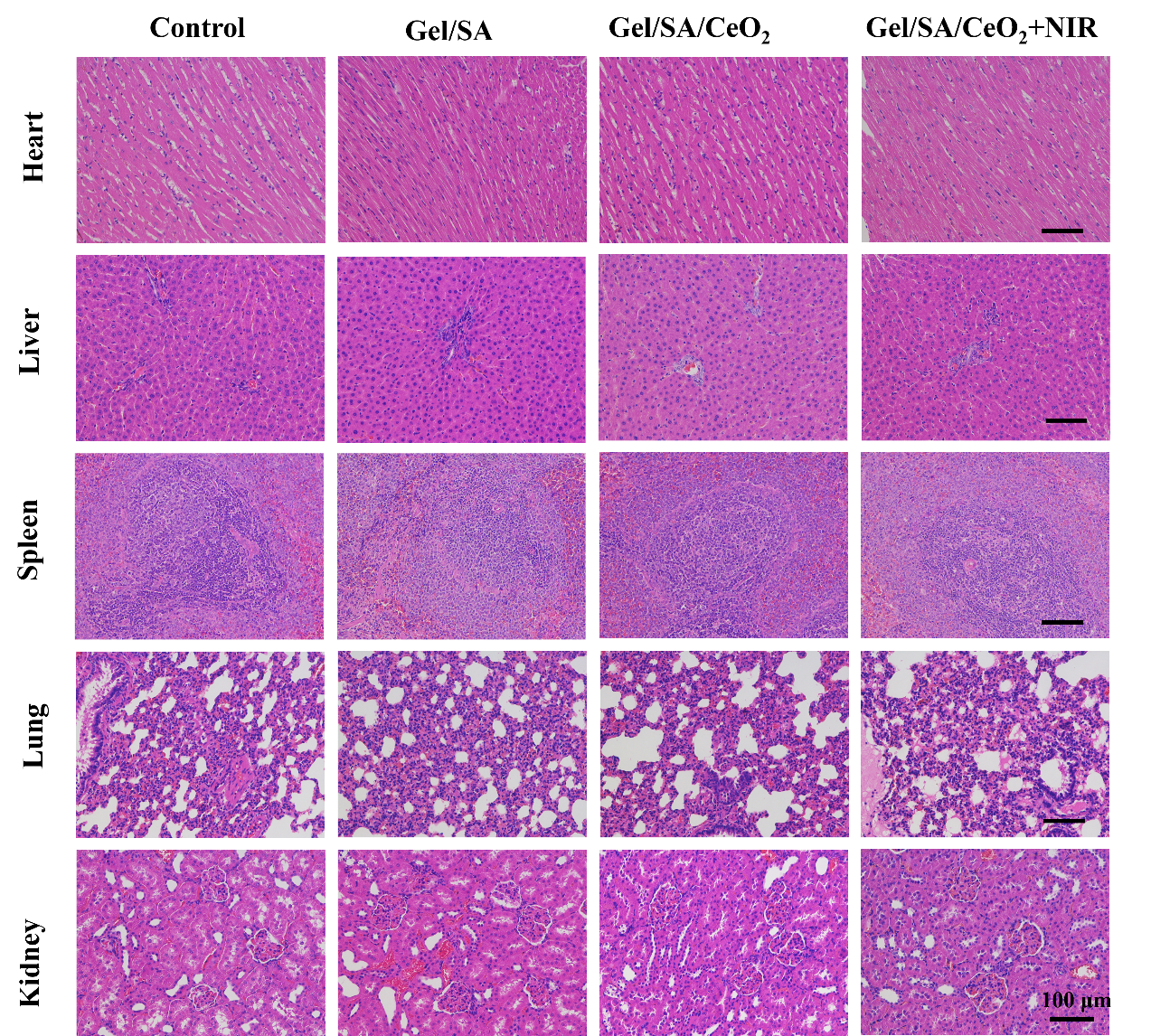
Fig. S7. H&E staining images of major organs (heart, liver, spleen, lung, kidney) of diabetic rats in different treatment groups after 14 days.

Fig. S8. The main blood biochemical indicators of diabetic rats in different treatment groups after 14 days
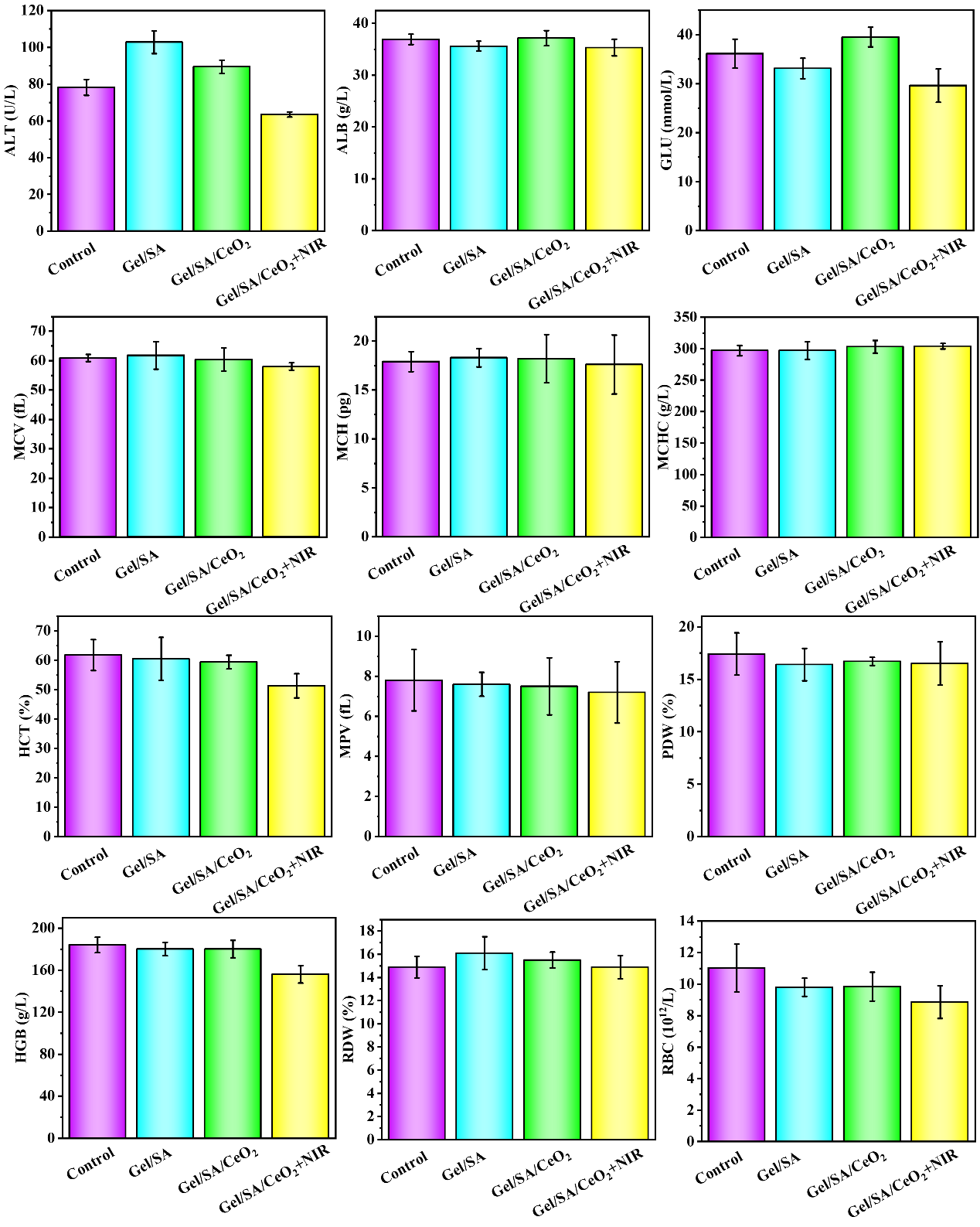
.


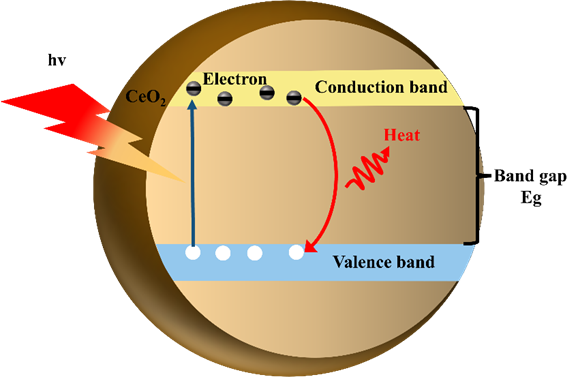


Fig. S9. Schematic diagram of light-to-heat conversion mechanism of CeO_2_.

According to the existing light-to-heat conversion mechanism of semiconductors and the photocatalytic mechanism of CeO2, we have explained the light-to-heat conversion mechanism of CeO2. Under the irradiation of light with an energy equal to or higher than the band gap of CeO2, electrons transition from the valence band to the conduction band, while holes are generated in the valence band to form electron-hole pairs. When the excited electrons return to the ground state and recombine with holes, it will cause local heating of the lattice, resulting in a photothermal effect. The schematic diagram is shown in the Fig.S3. (Elsevier, 2020: 109-167, DOI: 10.1016/B978-0-12-815661-2.00004-9; Nanoscale, 2021, 13, 8751, DOI: 10.1039/D1NR00323B).


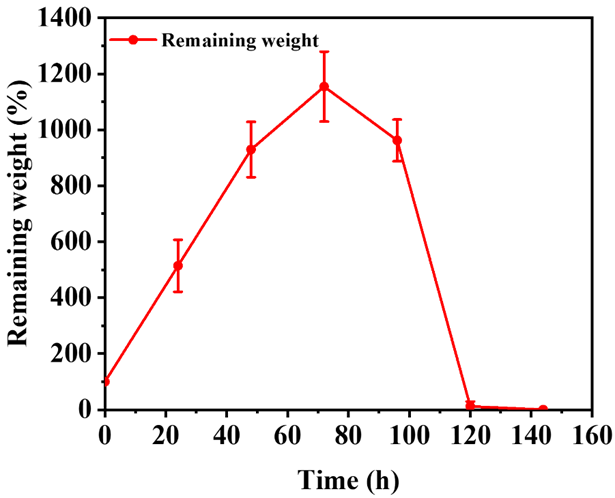


Fig. S10. Degradation curve of Gel/SA/CeO2-0.4% composite hydrogel in PBS.

We tested the degradation of Gel/SA/CeO_2_-0.4% hydrogel in PBS solution. It could be seen from the Fig. S10 that the hydrogel began to degrade on day 3 and completely degraded on day 6. From animal experiments, we found that the wound had scabbed and healed well on the third day, so the degradation did not affect the application of hydrogel in wound healing.


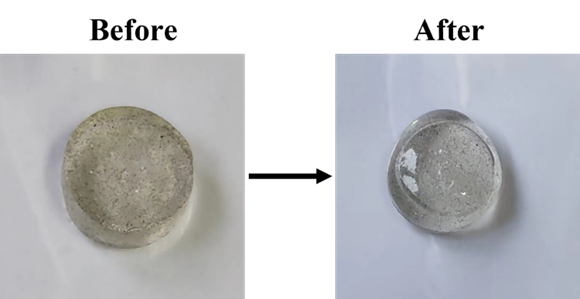


Fig. S11. The morphology pictures of the Gel/SA/CeO2-0.4% hydrogel before and after NIR irradiation


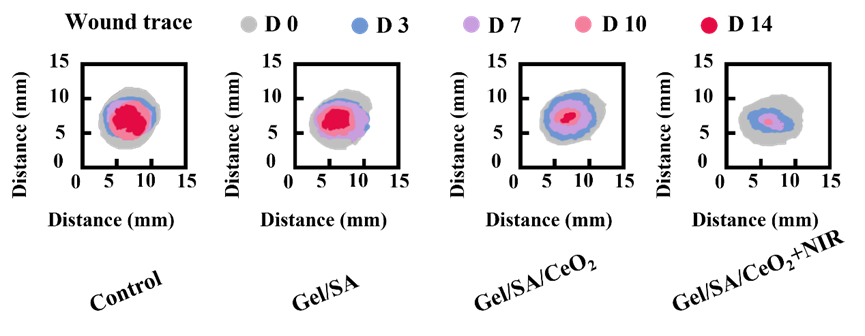


Fig. S12. Schematic diagram of wound healing

| mRNA | Forward primer | Reverse primer |
| --- | --- | --- |
| PDGF | 5’-GTCCGCCAACTTCCTGATCT-3' | 5’-TCCTCTAACCTCACCTGGACTT-3' |
| VEGF | 5’-GCAGATTATGCGGATCAAACC-3' | 5’-TTTCGTTTTTGCCCCTTTCC-3' |
| CD31 | 5’-GCTGTCACTGTCCCCTAAGA-3' | 5’-GTTAGGCAAAGGCTGAAGCT-3' |
| MMP9 | 5’-TACCACCTCGAACTTTGACA-3' | 5’-AGGGCGAGGACCATAGAG-3' |
| TGF-β1 | 5′-CGCTGCCCATCGTGTACTA-3′ | 5′-ACACAGAGATCCGCAGTCCT-3′ |
| GAPDH | 5′- GGGAAACTGTGGCGTGAT -3′ | 5′-GAGTGGGTGTCGCTGTTGA-3′ |

Table S1: Sequence of Primers for qRT-PCR.
